# SGLT2 inhibitor activates the STING/IRF3/IFN-β pathway and induces immune infiltration in osteosarcoma

**DOI:** 10.1038/s41419-022-04980-w

**Published:** 2022-06-03

**Authors:** Wei Wu, Zhenhao Zhang, Doudou Jing, Xin Huang, Dianyun Ren, Zengwu Shao, Zhicai Zhang

**Affiliations:** 1 Department of Orthopedics, Union Hospital, Tongji Medical College, Huazhong University of Science and Technology, Wuhan, 430022 China; 2 Department of Pancreatic Surgery, Union Hospital, Tongji Medical College, Huazhong University of Science and Technology, Wuhan, 430022 China

**Keywords:** Bone cancer, Cell signalling

## Abstract

SGLT2 (sodium-glucose cotransporter 2) is an important mediator of epithelial glucose transport and has been reported that SGLT2, robustly and diffusely expressed in malignant cancer cells, was overexpressed in various tumors, and inhibiting the SGLT2 expression significantly inhibited tumor progression. By blocking the functional activity of SGLT2, SGLT2 inhibitors have shown anticancer effects in several malignant cancers, including breast cancer, cervical cancer, hepatocellular cancer, prostate cancer, and lung cancer. However, the anticancer effect of SGLT2 inhibitors in osteosarcoma and the specific mechanism are still unclear. In the present study, we found that SGLT2 was overexpressed at the protein level in osteosarcoma. Furthermore, our results showed that the SGLT2 inhibitor significantly inhibited osteosarcoma tumor growth and induced infiltration of immune cells in vivo by upregulating STING expression and activating the IRF3/IFN-β pathway, which could attribute to the suppression of AKT phosphorylation. In addition, the combined treatment with SGLT2 inhibitor and STING agonist 2’3’-cGAMP exerted synergistic antitumor effects in osteosarcoma. Furthermore, the overexpression of SGLT2 at the protein level was correlated with the degradation of SGLT2 induced by TRIM21. This result demonstrated that SGLT2 is a novel therapeutic target of osteosarcoma, and that the SGLT2 inhibitor, especially in combination with 2’3’-cGAMP, is a potential therapeutic drug.

## Introduction

Osteosarcoma is a rare malignancy of bone, which primarily affects children and adolescents [1]. Surgery is the main treatment choice for osteosarcoma, but the survival is only ~15–17% for patients with osteosarcoma treated with surgery alone [2, 3]. Currently, combined therapies with surgical resection and chemotherapy are widely adopted for osteosarcoma patients and could cure ~70% of patients. However, osteosarcoma patients with distal metastasis or local relapse are resistant to conventional chemotherapy, and the survival of these patients remained virtually unchanged over the past 30 years, with an overall 5-year survival rate of only about 20% [3–5]. Given the poor prognosis of osteosarcoma patients, new therapeutic strategies are urgently needed. Bone has a highly specialized immune environment, and many immune signaling pathways are important in bone homeostasis [6]. Immune strategies show promise, which is the most substantial therapeutic advance in osteosarcoma in the past 10 years [6]. Immune therapy may represent the most promising potential therapeutic strategy for osteosarcoma patients.

SGLT2 (sodium-glucose cotransporter 2) is an important mediator of epithelial glucose transport and belongs to the SLC5A (solute carrier family 5) gene family [7]. SGLT2 is responsible for driving glucose and other nutrients into cells by harnessing the gradient of sodium ions across the plasma membrane [8]. It has been reported that SGLT2, robustly and diffusely expressed in malignant cancer cells, was overexpressed in various tumors, and inhibiting the SGLT2 expression significantly inhibited tumor progression [9]. However, the expression level and the specific biological role of SGLT2 in osteosarcoma still remains unclear.

Orally applied SGLT2 inhibitors that enter the blood and decrease renal reabsorption of glucose have been approved as antidiabetic drugs [10]. SGLT2 inhibitors decrease the concentration of plasma glucose by inhibiting the proximal tubular reabsorption of glucose in the kidney [11]. Mounting evidence suggests that SGLT2 inhibition may inhibit the growth of SGLT2-expressing carcinomas, including breast cancer [12], cervical cancer [13], hepatocellular cancer [14], prostate cancer, and lung cancer [15]. The antitumor and antiangiogenic activity of SGLT2 inhibitors could be attributed to their ability to inhibit the glucose uptake [16]. Therefore, SGLT2 inhibition may represent a novel therapeutic strategy for osteosarcoma patients.

In this study, we demonstrated that SGLT2 was overexpressed in osteosarcoma, and SGLT2 inhibition significantly inhibited the progression of osteosarcoma by inducing immune infiltration. Furthermore, the combined treatment with SGLT2 inhibitor and STING agonist synergistically inhibited the progression of osteosarcoma by further inducing immune infiltration. Thus, these results suggest that treatment with SGLT2 inhibitor, especially combined with STING (stimulator of interferon response cGAMP interactor) agonist, is a promising therapeutic strategy for osteosarcoma patients.

## Results

### SGLT2 is overexpressed at the protein level in osteosarcoma

**Fig. 1:**
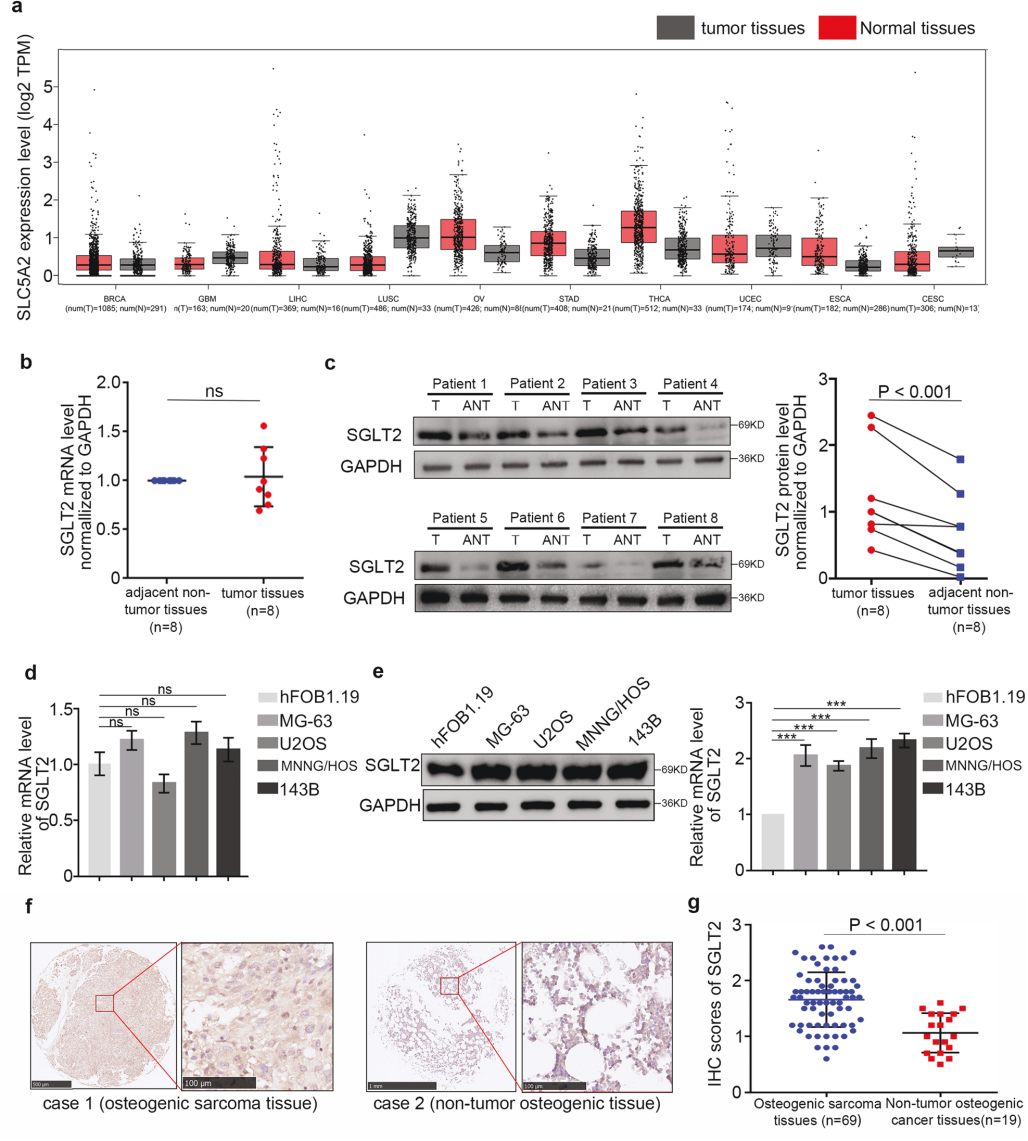
SGLT2 overexpression at the protein level in osteosarcoma. **a** Determination of the SGLT2 mRNA expression level by the GEPIA web tool. Boxplot analysis of the expression level showing log2 (TPM + 1) on a log-scale. RT-PCR analysis (**b**) and Western blot analysis (**c**) of the mRNA and protein expression of SGLT2 in eight paired osteosarcoma tumor tissues (T) and the matched adjacent normal tissues (ANT) of the same patient. GAPDH served as an internal reference. **d** RT-PCR analysis of the mRNA expression of SGLT2 in a normal osteogenic cell line (hFOB1.19 cell line) and four osteosarcoma cell lines (MG-63, U2OS, MNNG/HOS, and 143B cell lines). GAPDH served as an internal reference. **e** Western blot analysis of the protein expression of SGLT2 in a normal osteogenic cell line (hFOB1.19 cell line) and four osteosarcoma cell lines (MG-63, U2OS, MNNG/HOS, and 143B cell lines). Histogram to show the relative protein level of SGLT2 (*n* = 3). GAPDH served as an internal reference. **f** IHC images of SGLT2-stained Tissue Microarrays (TMAs). Scale bars are shown in the figure. **g** Dot plots of the IHC score of SGLT2 expression of TMA tissue sections (normal osteogenic specimens: *n* = 19, osteosarcoma specimens: *n* = 69, *p* < 0.001). Statistical analyses were performed using the D’Agostino and Pearson omnibus normality test.

To determine the expression level of SGLT2 in osteosarcoma tissues, the GEPIA web tool was searched, showing that SGLT2 was not overexpressed at the mRNA level in several malignant cancers (Fig. 1a). In addition, RT-PCR assay showed that the mRNA expression level of SGLT2 was also not upregulated in osteosarcoma patient specimens (Fig. 1b), while Western Blot analyses showed that the protein expression level of SGLT2 was significantly upregulated in osteosarcoma patient specimens (Fig. 1c). A similar effect was observed in a normal osteogenic cell line and osteosarcoma cell lines (Fig. 1d, e). Furthermore, IHC analysis showed that SGLT2 was significantly overexpressed in osteosarcoma tissues compared with normal osteogenic tissues (Fig. 1f, g). Taken together, this result indicated that SGLT2 was overexpressed in osteosarcoma at the protein level but not at the mRNA level, and the expression regulation of the overexpressed SGLT2 might occurred at the protein level but not at the mRNA level in osteosarcoma.

### TRIM21 induces SGLT2 degradation in osteosarcoma

**Fig. 2:**
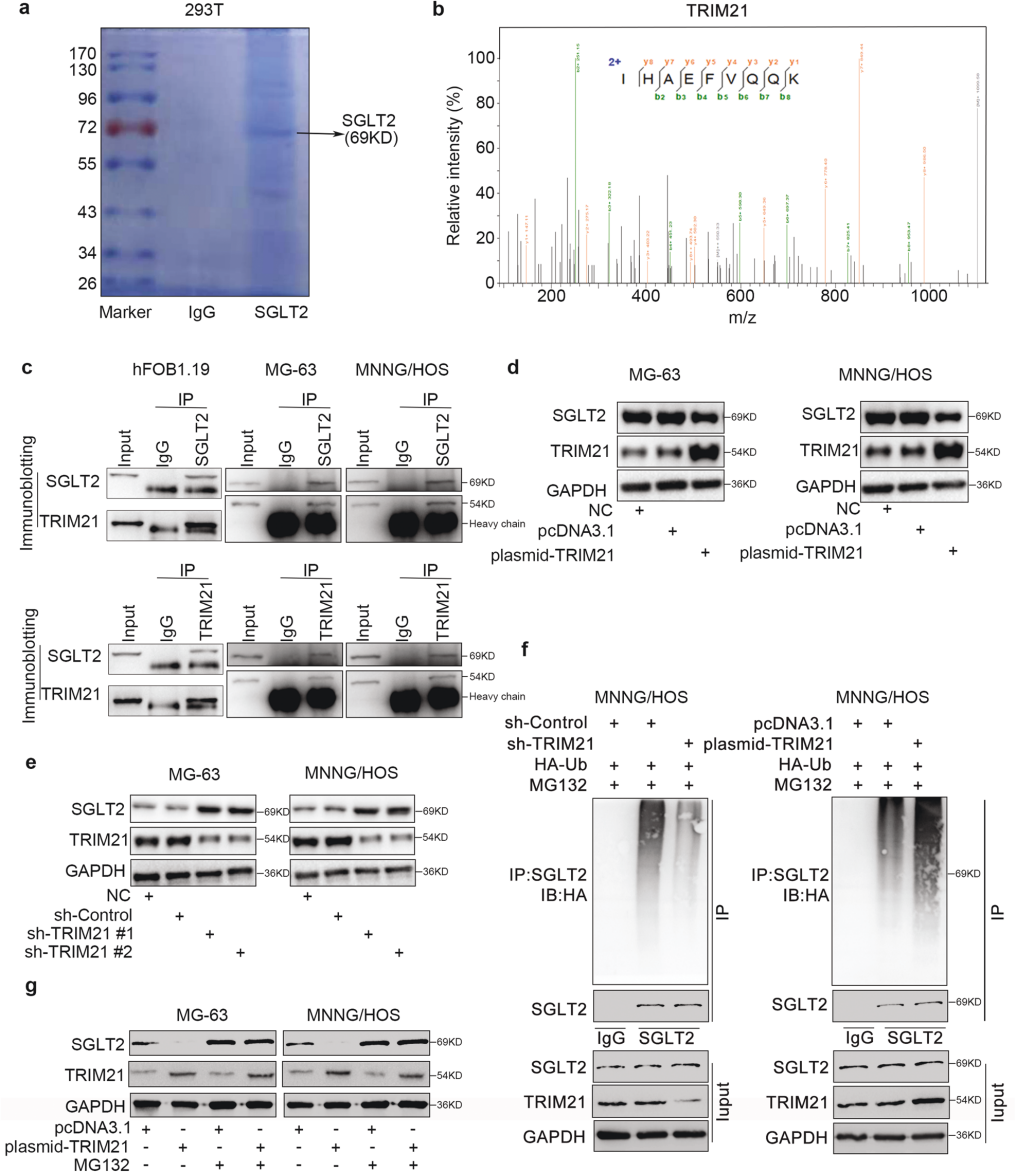
Induction of SGLT2 degradation by TRIM21 in osteosarcoma. LC-MS/MS analysis of the interaction between SGLT2 and TRIM21 (**a**) based on the detection of the peptide of TRIM21 (**b**). **c** Analysis of the interaction between SGLT2 and TRIM21 by co-immunoprecipitation. Western blot analysis of the protein expression levels of SGLT2 in TRIM21-overexpressed (**d**) and -silenced (**e**) MG-63 and MNNG/HOS cells. GAPDH served as an internal reference. **f** Western blot analysis in MG-63 cells transfected with sh-TRIM21 or TRIM21 plasmid for 48 h. Cells were treated with MG132 for 8 h before harvesting. **g** Western blot analysis of the protein expression levels of SGLT2 in TRIM21-overexpressed MG-63 and MNNG/HOS cells for 48 h. Cells were treated with MG132 for 8 h before harvesting. GAPDH served as an internal reference.

To further explore the mechanisms responsible for SGLT2 up-regulation in osteosarcoma, we performed LC-MS/MS and found that SGLT2 interacted with TRIM21 (tripartite motif containing 21; Fig. 2a, b). Moreover, SGLT2-TRIM21 interaction was confirmed by immunoprecipitation in normal osteogenic cell line and osteosarcoma cell lines (Fig. 2c). As a ubiquitination-related molecule, TRIM21 suppresses cancer progression by degrading various oncoproteins [17, 18]. In agreement with these reports, we found that TRIM21 overexpression significantly inhibited the protein expression of SGLT2, but not the mRNA expression of SGLT2 in osteosarcoma cells (Fig. 2d and Supplementary Fig. 1a), and TRIM21 silencing significantly induced the protein expression of SGLT2 in osteosarcoma cells (Fig. 2e). In addition, ubiquitin immunoprecipitation assay (Fig. 2f), Western blotting (Fig. 2g), and half-life determination assay (Supplementary Fig. 1b) indicated that TRIM21 silencing inhibited SGLT2 degradation, whereas TRIM21 overexpression enhanced SGLT2 degradation (Fig. 2f, g and Supplementary Fig. 1b). Furthermore, the Western Blot analyses showed that the protein expression level of TRIM21 was significantly downregulated in osteosarcoma patient specimens (Supplementary Fig. 1c), consistent with the overexpression of SGLT2 in osteosarcoma patient specimens. Therefore, the overexpression of SGLT2 at the protein level could be attributed to the SGLT2 degradation induced by TRIM21 in osteosarcoma.

### SGLT2 inhibitor inhibits osteosarcoma progression by inducing immune cell infiltration

**Fig. 3:**
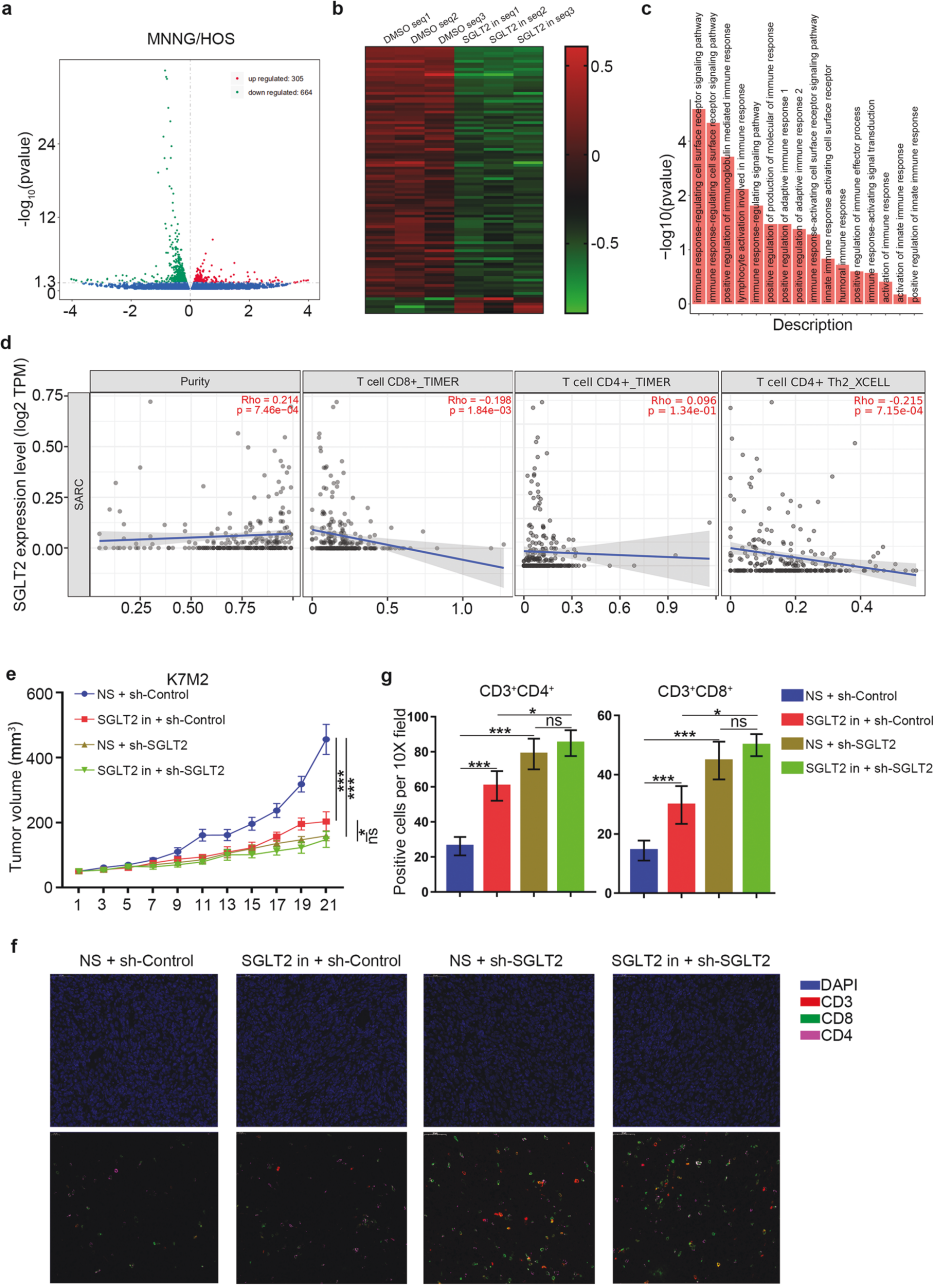
Inhibition of osteosarcoma progression due to the induction of immune cell infiltration by the SGLT2 inhibitor. MNNG/HOS cells treated with or without SGLT2 inhibitor (canagliflozin, 1 uM) for 24 h were harvested for RNA sequencing analysis, and Volcano plot (**a**) and heatmap (**b**) showed the differentially expressed genes in MNNG/HOS cells treated with or without SGLT2 inhibitor. Green points represent downregulated genes, while red points represent upregulated genes. **c** Histogram of the GO-enriched pathways related with immune infiltration in the SGLT2 inhibitor treatment group. **d** TIMER web tool was used to search the relationship of the mRNA expression level of SGLT2 and the infiltration level of CD8^+^ T cells, CD4^+^ T cells, and CD4^+^ Th2 cells in osteosarcoma. **e** K7M2 tumor growth curves (*n* = 5/group) of different groups (***p < 0.001). NS normal saline. **f**, **g** Immunofluorescence staining analysis of the percentage of CD3^+^CD4^+^ and CD3^+^CD8^+^ T cells infiltrated in K7M2 tumors (**f**). Data are presented as the mean ± SD (**g**, *n* = 5/group, ***p < 0.001).

To explore the specific mechanism underlying the regulation of osteosarcoma progression by the SGLT2 inhibitor (canagliflozin, 1 uM), we performed RNA sequence analysis and determined 664 downregulated differentially expressed genes and 305 upregulated differentially expressed genes in SGLT2 inhibitor-treated osteosarcoma cells (Fig. 3a, b). In addition, Gene Ontology enrichment analysis showed that immune response-related pathways were significantly enriched in the SGLT2 inhibitor treatment group (Fig. 3c). In agreement with this result, by searching the TIMER web tool, we found that the mRNA expression level of SGLT2 was negatively correlated with the infiltration level of immune cells in sarcoma, including CD8^+^ T cells, CD4^+^ T cells, and CD4^+^ Th2 cells (Fig. 3d). To verify these results, we established a syngeneic tumor model, which revealed that the SGLT2 inhibitor or SGLT2 silencing both significantly inhibited the tumor growth (Fig. 3e) and significantly increased the number of infiltrated immune cells, including CD4^+^ and CD8^+^ lymphocyte cells (Fig. 3f, g and Supplementary Fig. 2a). However, SGLT2 inhibitor had no effect in inhibiting the tumor growth and increasing the number of infiltrated immune cells in SGLT2 silenced osteosarcoma, which indicated that the anti-osteosarcoma effect SGLT2 inhibitor depended on the expression of SGLT2 (Fig. 3e–g). Besides, the circulating and splenic CD4^+^ and CD8^+^ lymphocyte cell numbers was also increased after treatment of SGLT2 inhibitor, although not significantly, which could attribute to the stimulated superior systemic immune response (Supplementary Fig. 2b, c). Therefore, our result indicated that SGLT2 inhibitor treatment inhibited the progression of osteosarcoma, which might attribute to the inducing immune cell infiltration.

### SGLT2 inhibitor induces STING expression in osteosarcoma cells

**Fig. 4:**
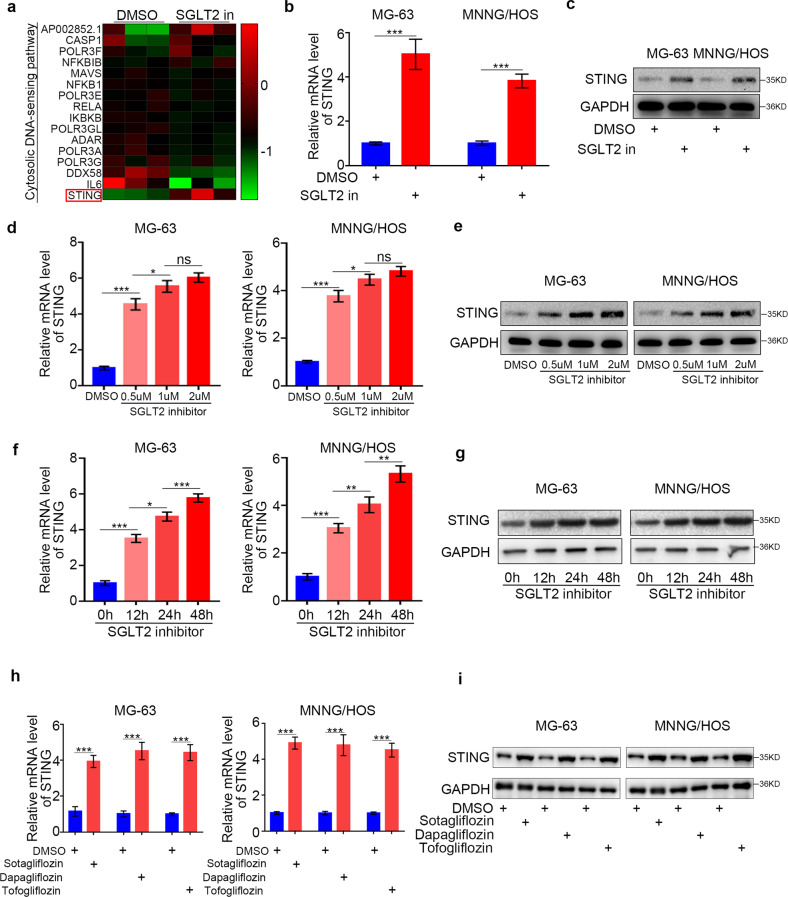
SGLT2 inhibitor-induced STING expression in osteosarcoma cells. **a** Heatmap of the SGLT2 inhibitor treatment-related genes that participated in the cytosolic DNA-sensing pathway in MNNG/HOS cells. RT-PCR (**b**) and Western blot (**c**) analyses of the mRNA and protein expression levels of STING in MG-63 and MNNG/HOS cell lines treated with DMSO or SGLT2 inhibitor (canagliflozin, 1 uM) for 24 h. GAPDH served as an internal reference. RT-PCR (**d**) and Western blot (**e**) analyses of the mRNA and protein expression levels of STING in MG-63 and MNNG/HOS cell lines treated with DMSO or SGLT2 inhibitor (canagliflozin; 0.5, 1, or 2 uM) for 24 h. Data are presented as the mean ± SD of three independent experiments (ns not significant; *p < 0.05; ***p < 0.001). RT-PCR (**f**) and Western blot (**g**) analyses of the mRNA and protein expression levels of STING in MG-63 and MNNG/HOS cell lines treated with SGLT2 inhibitor (canagliflozin, 1 uM) for 0, 12, 24 or 48 h. Data are presented as the mean ± SD of three independent experiments (*p < 0.05; **p < 0.01; ***p < 0.001). RT-PCR (**h**) and Western blot (**i**) analyses of the mRNA and protein expression levels of STING in MG-63 and MNNG/HOS cell lines treated with Sotagliflozin (200 nM), Dapagliflozin (100 nM), and Tofogliflozin (100 nM) for 24 h, respectively. Data are presented as the mean ± SD of three independent experiments (*p < 0.05; **p < 0.01; ***p < 0.001).

To determine the specific mechanism by which SGLT2 inhibitor treatment promoted the infiltration of immune cells in osteosarcoma, we performed KEGG signaling pathway enrichment analysis based on RNA sequence data, showing that the cytosolic DNA-sensing pathway was significantly enriched, and the expression of STING was significantly upregulated in the SGLT2 inhibitor (Canagliflozin, 1.0 uM [19, 20]) treatment group (Fig. 4a and Supplementary Table 3). Then, we hypothesized that SGLT2 inhibitor treatment could up-regulate the expression of STING in osteosarcoma cells, which was verified by RT-PCR and Western blot analyses (Fig. 4b, c and Supplementary Fig. 3a). In addition, SGLT2 inhibitor treatment induced STING up-regulation at the mRNA and protein levels in a dose-dependent manner (Fig. 4d, e and Supplementary Fig. 3b). Besides, SGLT2 inhibitor treatment could also induce STING up-regulation at the mRNA and protein levels in a time-dependent manner (Fig. 4f, g and Supplementary Fig. 3c). The result showed that the significantly up-regulation of STING mRNA expression first appeared after treatment of SGLT2 inhibitor for 4 h (Supplementary Fig. 3d), whereas the significantly up-regulation of STING protein expression first appeared after treatment of SGLT2 inhibitor for 8 h (Supplementary Fig. 3e, f). Furthermore, with the treatment of cycloheximide (CHX) to inhibit the protein synthesis, mRNA expression of STING still significantly upregulated in osteosarcoma cells treated with SGLT2 inhibitor, which indicated that SGLT2 inhibitor-induced STING expression in a direct way (Supplementary Fig. 3g). Consistently, the expression of STING in osteosarcoma cells was also upregulated after treatment with other SGLT2 inhibitors, including Sotagliflozin, Dapagliflozin, and Tofogliflozin (Fig. 4h, i and Supplementary Fig. 3h). Furthermore, in SGLT2 silenced osteosarcoma cells, SGLT2 inhibitor could not induce STING expression (Supplementary Fig. 3i, j). Taken together, all the results suggested that the SGLT2 inhibitor induces the expression of STING via blocking SGLT2 in osteosarcoma cells.

### SGLT2 inhibitor activates the STING/IRF3/IFN-β pathway in osteosarcoma cells

**Fig. 5:**
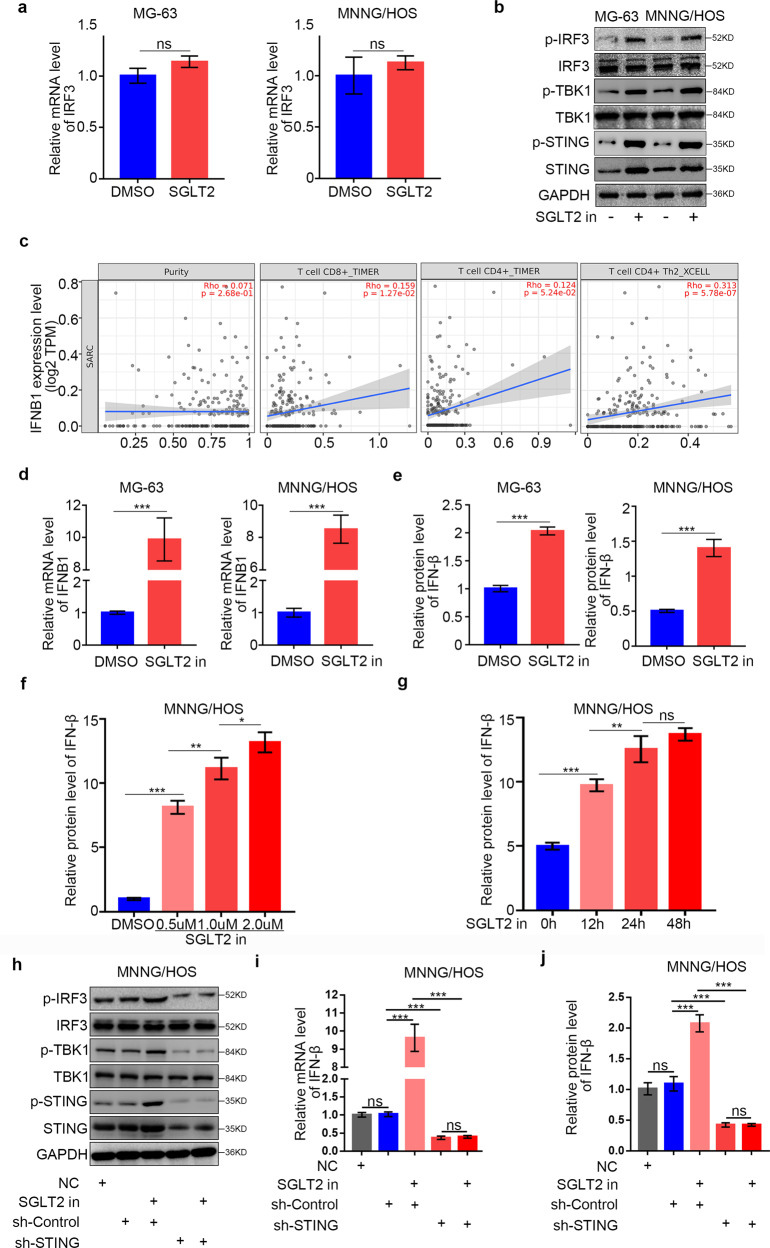
Activation of the STING/IRF3/IFN-β pathway in osteosarcoma cells by the SGLT2 inhibitor. RT-PCR (**a**) and Western blot (**b**) analyses of the mRNA and protein expression levels of specific genes in MG-63 and MNNG/HOS cell lines with or without treatment with SGLT2 inhibitor (canagliflozin, 1 uM) for 48 h. GAPDH served as an internal reference. **c** TIMER web tool was used to search the relationship of the mRNA expression level of IFNB1 and the infiltration of CD4^+^/CD8^+^ T cells in osteosarcoma. RT-PCR (**d**) and ELISA (**e**) analyses of the mRNA and protein expression levels of IFN-β in MG-63 and MNNG/HOS cell lines with or without treatment with SGLT2 inhibitor (canagliflozin, 1 uM) for 48 h. Data are presented as the mean ± SD of three independent experiments (***p < 0.001). **f** ELISA analysis of the protein expression level of IFN-β in MNNG/HOS cell lines with or without treatment with SGLT2 inhibitor (canagliflozin, 0, 0.5, 1, or 2 uM) for 48 h. Data are presented as the mean ± SD of three independent experiments (ns not significant; *p < 0.05; ***p < 0.001). **g** ELISA analysis of the protein expression level of IFN-β in MNNG/HOS cell lines with or without treatment with SGLT2 inhibitor (canagliflozin, 1 uM) for 0, 12, 24 or 48 h. Data are presented as the mean ± SD of three independent experiments (*p < 0.05; **p < 0.01; ***p < 0.001). **h** Western Blot analyses of the protein expression levels in STING silenced MG-63 and MNNG/HOS cell lines with or without treatment with SGLT2 inhibitor (canagliflozin, 1 uM) for 48 h. RT-PCR (**i**) and ELISA (**j**) analyses of the mRNA and protein expression levels of IFN-β in STING silenced MG-63 and MNNG/HOS cell lines with or without treatment with SGLT2 inhibitor (canagliflozin, 1 uM) for 48 h. Data are presented as the mean ± SD of three independent experiments (***p < 0.001).

STING pathway activation has been shown to induce immune cell activation by activating the IRF3 (interferon regulatory factor 3)/IFN-β (interferon beta) pathway [21]. Here, we explored the ability of the SGLT2 inhibitor to activate the STING/IRF3 pathway in osteosarcoma cells. We found that SGLT2 inhibitor treatment significantly increased the phosphorylation levels of STING, IRF3, and TBK1 compared with the control group, indicating activation of the STING/IRF3 pathway (Fig. 5a, b). By searching TIMER web tool, we also found that the IFNB1 mRNA expression level was positively correlated with the infiltration levels CD8^+^ T cells, CD4^+^ T cells, and CD4^+^ Th2 cells in sarcoma (Fig. 5c). Furthermore, SGLT2 inhibitor treatment in osteosarcoma cells induced IFN-β expression at the mRNA and protein levels (Fig. 5d, e) in both dose- and time-dependent manner (Fig. 5f, g). Finally, STING silencing significantly inhibited the up-regulation of p-IRF3 and IFN-β expression induced by SGLT2 inhibitor treatment in osteosarcoma cells (Fig. 5h–j), which suggesting that the SGLT2 inhibitor activated the STING/IRF3/IFN-β pathway in osteosarcoma cells.

### SGLT2 inhibitor treatment and cGAMP treatment synergistically activate the STING/IRF3/IFN-β pathway in osteosarcoma cells

**Fig. 6:**
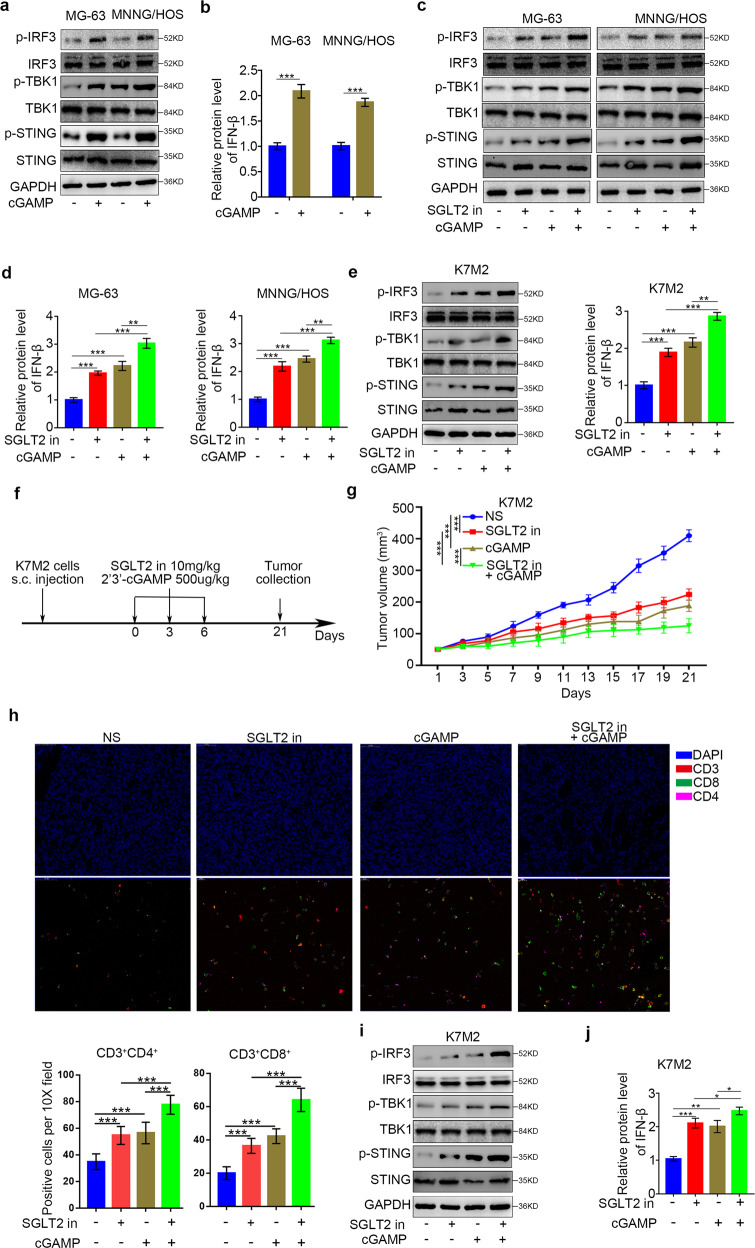
Synergistic activation of the STING/IRF3/IFN-β pathway in osteosarcoma cells by treatment with SGLT2 inhibitor and cGAMP. **a** Western blot analysis of the protein expression levels in MG-63 and MNNG/HOS cell lines with or without treatment with 2’3’-cGAMP (150 nM) for 48 h. GAPDH served as an internal reference. **b** ELISA analysis of the protein expression levels of IFN-β in MG-63 and MNNG/HOS cell lines with or without treatment with 2’3’-cGAMP (150 nM) for 48 h. Data are presented as the mean ± SD of three independent experiments (***p < 0.001). **c** Western blot analysis of the protein expression levels in MG-63 and MNNG/HOS cell lines treated with canagliflozin (1 uM), 2’3’-cGAMP (150 nM), or a combination of canagliflozin (1 uM) and 2’3’-cGAMP (150 nM) for 48 h. **d** ELISA analysis of the protein expression level of IFN-β in MG-63 and MNNG/HOS cell lines treated with canagliflozin (1 uM), 2’3’-cGAMP (150 nM), or a combination of canagliflozin (1 uM) and 2’3’-cGAMP (150 nM) for 48 h. GAPDH served as an internal reference (**p < 0.01; ***p < 0.001). **e** Western blot and ELISA analyses of the protein expression levels in the K7M2 cell line treated with canagliflozin (1 uM), 2’3’-cGAMP (150 nM), or a combination of canagliflozin (1 uM) and 2’3’-cGAMP (150 nM) for 48 h. GAPDH served as an internal reference. **f** Schematic diagram of the procedure of the in vivo experiments; the doses of cGAMP and SGLT2 inhibitor (canagliflozin) are indicated above. **g** K7M2 tumor growth curves of different groups (*n* = 5/group; ***p < 0.001). **h** Immunofluorescence analysis of the percentage of infiltrated CD3^+^CD4^+^ and CD3^+^CD8^+^ T cells per 10X field. Data are presented as the mean ± SD (*n* = 5/group; ***p < 0.001). **i** Western blot analysis of the protein expression levels in the collected tumors. **j** ELISA analysis of the protein expression level of IFN-β in in the collected tumors. GAPDH served as an internal reference (*p < 0.05; **p < 0.01; ***p < 0.001).

Studies have shown that 2’3’-cGAMP significantly activated immune cell as a STING pathway agonist [22–24]. Study has shown that Treatment to elevate STING expression enhances the cancer immunotherapy effect of cGAMP in mice [25]. Having established that the SGLT2 inhibitor activates the STING/IRF3/IFN-β pathway in osteosarcoma cells, we investigated the potential synergistic effects of the STING pathway agonist 2’3’-cGAMP and the SGLT2 inhibitor in osteosarcoma. First of all, we verified that 2’3’-cGAMP also increased the expression level of p-STING, p-IRF3, p-TBK1 and IFN-*β* in osteosarcoma cells (Fig. 6a, b). Furthermore, the expression levels of p-STING, p-IRF3, p-TBK1, and IFN-*β* were further increased when the cells were simultaneously treated with SGLT2 inhibitor and 2’3’-cGAMP (Fig. 6c, d and Supplementary Fig. 4a).

Next, we investigated the effects of the SGLT2 inhibitor on T cell infiltration in vivo and confirmed that the SGLT2 inhibitor activated the STING/IRF3/IFN-β pathway in murine K7M2 tumor cells. Moreover, the combination of SGLT2 inhibitor and 2’3’-cGAMP further increased the p-STING, p-IRF3, p-TBK1, and IFN-*β* expression levels compared with SGLT2 inhibitor or 2’3’-cGAMP treatment alone (Fig. 6e and Supplementary Fig. 4b). After the subcutaneous injection of K7M2 cells into the flanks of C57BL immune-proficient mice, we treated the mice with SGLT2 inhibitor and 2’3’-cGAMP (Fig. 6f). The combination of SGLT2 inhibitor and 2’3’-cGAMP showed a synergistic effect in the inhibition of tumor growth (Fig. 6g). Moreover, treatment with SGLT2 inhibitor or 2’3’-cGAMP alone or in combination promoted tumor infiltration by CD45^+^CD4^+^ and CD45^+^CD8^+^ T cells (Fig. 6h and Supplementary Fig. 4c), which may explain their synergistic effect in suppressing tumor growth. In addition, we confirmed that the combination of SGLT2 inhibitor and 2’3’-cGAMP had a synergistic effect on the activation of the STING/IRF3/IFN-β pathway in vivo (Fig. 6i, j and Supplementary Fig. 4d).

Furthermore, we also perform the syngeneic tumor model experiments with murine K7M2 tumor cells infected with sh-Control or sh-STING (Supplementary Fig. 4e). The SGLT2 inhibitor treatment showed a significantly antitumor effect in STING normal expressed K7M2 tumors, whereas a limited antitumor effect in STING silenced K7M2 tumors (Supplementary Fig. 4f, g). In addition, Moreover, treatment with SGLT2 inhibitor promoted tumor infiltration by CD45^+^CD4^+^ and CD45^+^CD8^+^ T cells in STING normal expressed K7M2 tumors, while not in in STING silenced K7M2 tumors (Supplementary Fig. 4h). These results suggested that the SGLT2 inhibitor suppressed osteosarcoma progression and promoted T cell infiltration in vivo, especially when mice were simultaneously treated with 2’3’-cGAMP.

### SGLT2 inhibitor increases the expression level of STING via repressing the AKT pathway in osteosarcoma

**Fig. 7:**
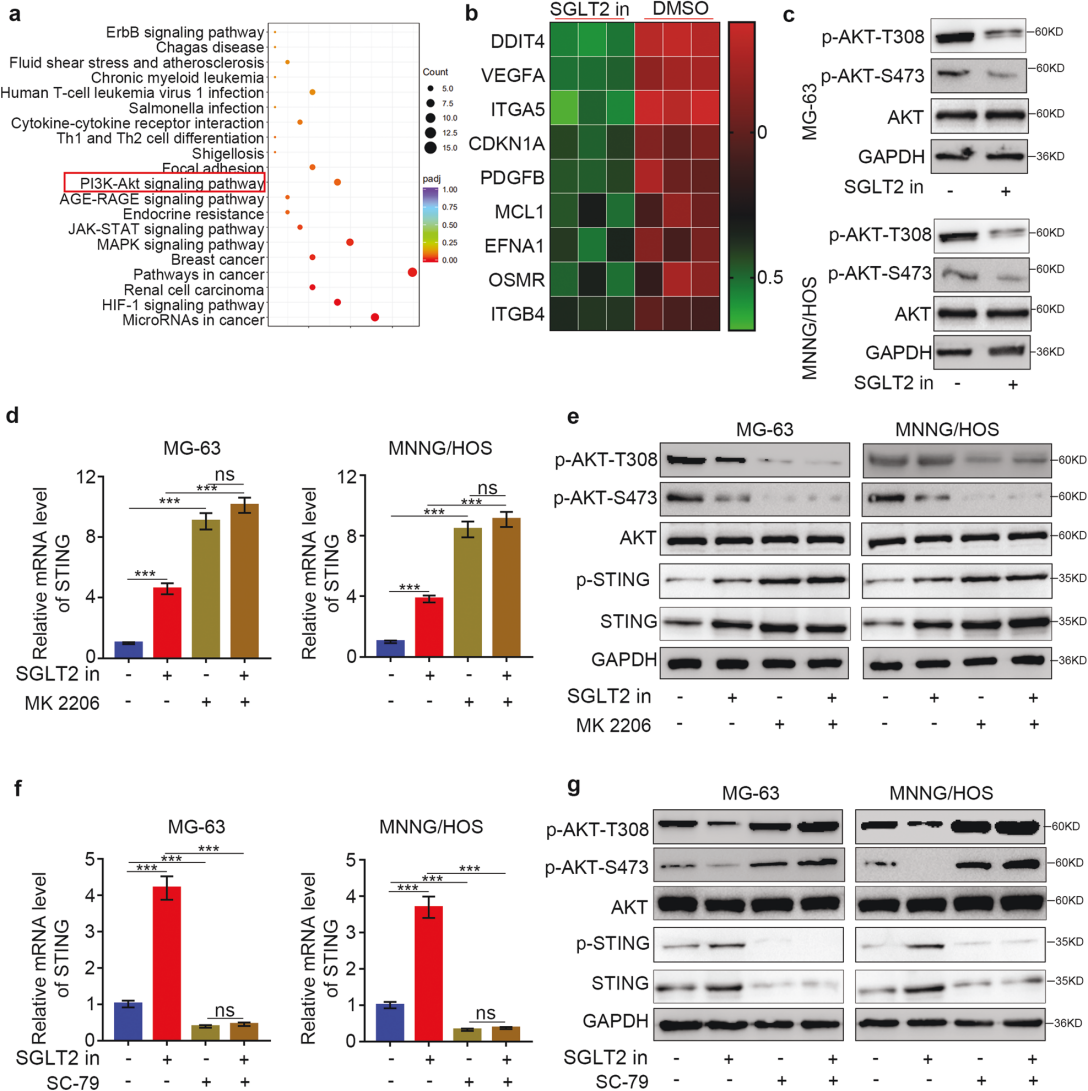
SGLT2 inhibitor increased the expression level of STING via repressing the AKT pathway in osteosarcoma. **a** Dot plot of the KEGG enrichment pathways in the SGLT2 inhibitor treatment group. **b** Heatmap of the SGLT2 inhibitor treatment-related genes that participated in the PI3K-AKT pathway in MNNG/HOS cells. **c** Western blot analysis of the protein expression levels of AKT and p-AKT in MG-63 and MNNG/HOS cell lines treated with canagliflozin (1 uM) for 24 h. GAPDH served as an internal reference. RT-PCR analysis (**d**) and Western blot analysis (**e**) of the mRNA and protein expression levels in MG-63 and MNNG/HOS cell lines treated with canagliflozin (1 uM), MK2206 (10 μM), or the combination of canagliflozin (1 uM) and MK2206 (10 μM). Data are presented as the mean ± SD of three independent experiments (ns not significant; ***p < 0.001). RT-PCR analysis (**f**) and Western blot analysis (**g**) of the mRNA and protein expression levels of specific genes in MG-63 and MNNG/HOS cell lines treated with canagliflozin (1 uM), SC-79 (20 μM), or the combination of canagliflozin (1 uM) and SC-79 (20 μM) for 24 h. Data are presented as the mean ± SD of three independent experiments (ns not significant; ***p < 0.001).

The phosphorylation level of AKT has been reported to be negatively correlated with the STING pathway [26]. Moreover, KEGG signaling pathway enrichment analysis showed that the PI3K-AKT pathway was significantly enriched in the SGLT2 inhibitor treatment group (Fig. 7a, b). Therefore, we hypothesized that SGLT2 inhibitor treatment could regulate the expression of STING in osteosarcoma via inhibition of the AKT pathway. Firstly, our data indicated that treatment with SGLT2 inhibitor decreased the phosphorylation level of AKT in osteosarcoma cells (Fig. 7c). Secondly, the increasing expression of STING (Fig. 7d, e) and IFN-β (Supplementary Fig. 5a) induced by the SGLT2 inhibitor was enhanced by the inhibition of AKT signaling by the AKT inhibitor MK2206, which inhibited the auto-phosphorylation effect of AKT on Thr308 and Ser473 sites. Furthermore, the increasing expression of STING induced by the SGLT2 inhibitor was attenuated by the treatment of AKT agonist SC-79 (Fig. 7f, g). These data indicated that SGLT2 inhibitor treatment induced STING gene expression in a direct way, via repressing the phosphorylation level of AKT. Thus, our results suggested that SGLT2 inhibitor treatment could increase the expression level of STING by repressing the activation of the AKT pathway.

## Discussion

As mentioned above, SGLT2 was overexpressed in malignant cancers, including pancreatic and prostate adenocarcinomas [9], lung cancer [27], and breast cancer [28], and the overexpression of SGLT2 significantly promoted the proliferation, migration, and invasion abilities in malignant cancers [12, 13]. In our study, we found that SGLT2 was overexpressed at the protein level in osteosarcoma, which indicated that SGLT2 is a novel therapeutic target in osteosarcoma.

It has been reported that specific SGLT2 inhibitors could block the functional activity of SGLT2 [9]. Kaji et al. found that the SGLT2 inhibitor canagliflozin attenuated liver cancer cell growth and the angiogenic activity by inhibiting the uptake of glucose [16]. Xie et al. reported that the SGLT2 inhibitor modulated SHH expression by activating AMPK to inhibit the migration and induce the apoptosis of cervical carcinoma cells [13]. SGLT2 inhibitors also attenuated breast cancer cell proliferation via membrane hyperpolarization and mitochondrial membrane instability [28]. However, no anticancer effect and specific mechanism of SGLT2 inhibitors in osteosarcoma have been reported yet. In our study, we found that the SGLT2 inhibitor significantly inhibited osteosarcoma tumor growth in vivo, which could be attributed to the up-regulation of STING expression and activation of the IRF3/IFN-β pathway induced by the SGLT2 inhibitor. AKT signaling has been previously shown to negatively regulate the STING pathway [29]. We also confirmed the link between SGLT2 inhibitor, AKT, and STING in osteosarcoma: the SGLT2 inhibitor enhanced STING expression by suppressing AKT phosphorylation and subsequent activation. This finding unveiled a previously unknown mechanism involved in the activation of the SGLT2 inhibitor-mediated STING pathway.

Previous studies had reported the non-interferon responses downstream of STING, especially for nuclear factor-κB (NF-κB) activation have not been mentioned but constitute an important part of the functions of STING. Abe et al. found that Cytosolic DNA-mediated, STING-dependent proinflammatory gene induction necessitates canonical NF-κB activation through TBK1 [30]. Once activated, the STING pathway induces both type I interferon (IFN) expression and NF-κB-mediated cytokine production [31]. The induction of type I interferons through the transcription factor interferon regulatory factor 3 (IRF3) is considered a major outcome of stimulator of interferon genes (STING) activation that drives immune responses against DNA viruses and tumors. However, STING activation can also trigger other downstream pathways such as NF-κB signaling. Studies had showed that STING can function independently of type I interferons [32]. The study of Balka et al. showed that TBK1 and IKKε act redundantly to mediate STING-induced NF-κB responses in myeloid cells. Furthermore, the tumor induces STING-mediated cell death in T cells to evade immune control that is in part mediated by IFN-independent activities of STING [33]. Consistently, Yamashiro et al. also found that interferon-independent functions of STING mediate STING-dependent antiviral responses in vivo [34]. Therefore, in addition to interferon signaling pathway, STING could also induce other downstream pathways such as nuclear factor κB (NF-κB) signaling, which could also be the potential therapeutic target in malignant cancer and need further research to conducted.

The STING agonist 2’3’-cGAMP is a second messenger of the STING pathway [22] that binds directly to and activates STING [23, 24] and has been shown to modulate the tumor microenvironment and reduce the tumor burden in cancer [35]. Study has shown that Treatment to elevate STING expression enhances the cancer immunotherapy effect of cGAMP in mice [25]. Importantly, our study showed that 2’3’-cGAMP promoted the infiltration of CD4^+^ and CD8^+^ T cells in osteosarcoma. Given the up-regulation of STING expression induced by the SGLT2 inhibitor, we also found that the combined treatment with SGLT2 inhibitor and 2’3’-cGAMP further promoted the infiltration of CD4^+^ and CD8^+^ T cells, exerting synergistic antitumor effects in osteosarcoma. Taken together, our results indicated that the SGLT2 inhibitor is a novel potential therapeutic drug against osteosarcoma, especially in combination with 2’3’-cGAMP.

The PD-1/PD-L1 immune checkpoint blockade allows to potentiate antitumor immunity by inhibiting immunosuppressive signals from co-inhibitory molecules, and this has achieved great success in many malignant tumors [36–40]. The interaction between PD-L1 on tumor cells and PD-1 on T-cells inhibits the biological functioning of antigen-specific CD8^+^ T cells and conduces cancer cells to escape immune destruction [41, 42]. Previous studies had shown that the PD-1 pathway blockade is beneficial to the antitumor effect of STING activation [43, 44]. We also investigated the anti-osteosarcoma effect of combined treatment of SGLT2 inhibitor and immunotherapy (anti-PD-1). And the result showed that SGLT2 inhibitor treatment further inhibited the tumor growth and promoted the infiltration of CD4+ and CD8+ T cells induced by immunotherapy (anti-PD-1) therapy in osteosarcoma (Supplementary Fig. 6). Taken together, our results indicated that the the combined treatment of SGLT2 inhibitor and immunotherapy (anti-PD-1) might be a novel potential therapeutic strategy for osteosarcoma.

TRIM21 is a RING finger domain-containing ubiquitin E3 ligase. It has been reported that the expression of TRIM21 was decreased in several malignant cancers, including colitis-associated cancer [17] and breast cancer [18]. TRIM21 silencing promotes cancer cell proliferation, migration, and the ability of anti-apoptosis [45, 46] and regulates epithelial carcinogenesis [17]. In addition, the expression of TRIM21 is elevated in autoimmune diseases, and TRIM21 plays an important role in immune activation during pathogen infection, but only little is known about its inherent cellular function [47]. Moreover, TRIM21 plays a key role in intracellular protection by targeting invading viruses for destruction and alerting the immune system [48]. Previous studies have shown that overexpression of TRIM21 resulted in less production of interferon-β (IFN-β) in response to intracellular dsDNA, which could be attributed to the increased degradation of DDX41 [49] and IFI16 [50]. In contrast, our study indicated that the overexpression of TRIM21 resulted in an increased degradation of SGLT2, which induced the expression of STING and the activation of the IRF3/IFN-β pathway in osteosarcoma. Previously studies had showed that TRIM21 expression was decreased in several cancers at both the mRNA and protein levels in comparison to that in nontumorous tissues [51, 52]. However, whether the expression level of TRIM21 was decreased in osteosarcoma tissues compared with non-osteosarcoma still be unclear, which need further researched. However, the specific role of TRIM21 in the STING/IRF3/IFN-β pathway still needs to be verified in further research.

## Conclusion

**Fig. 8:**
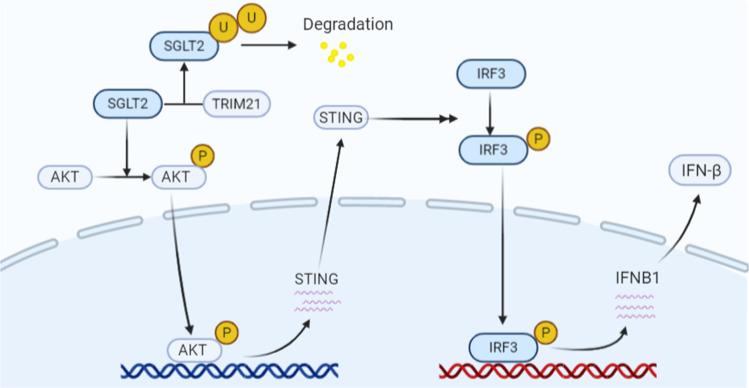
In osteosarcoma, TRIM21 induced the degradation of SGLT2 and the overexpression of SGLT2 at the protein level. The SGLT2 inhibitor significantly upregulated the STING expression and activated the STING/IRF3/IFN-β pathway. Therefore, the combined treatment with SGLT2 inhibitor and STING agonist 2’3’-cGAMP exerted synergistic antitumor effects in osteosarcoma, as SGLT2 inhibitor 2’3’-cGAMP activate the STING/IRF3/IFN-β pathway via different mechanisms.

In our study, we found that SGLT2 was overexpressed at the protein level in osteosarcoma, and we demonstrated that the SGLT2 inhibitor significantly inhibited osteosarcoma tumor growth and induced infiltration of immune cells in vivo by upregulating STING expression, which could be contributed to the suppression of AKT phosphorylation (Fig. 8). Furthermore, the combined treatment with SGLT2 inhibitor and STING agonist 2’3’-cGAMP exerted synergistic antitumor effects in osteosarcoma due to the activation of the STING/IRF3/IFN-β pathway via different mechanisms. Moreover, the overexpression of SGLT2 at the protein level was correlated with the degradation of SGLT2 induced by TRIM21. Taken together, our results indicate that SGLT2 inhibitor, especially combined with 2’3’-cGAMP, is a novel potential therapeutic drug against osteosarcoma.

## Materials and methods

### Cell culture and chemicals

Four human osteosarcoma cell lines (MNNG/HOS, MG-63, 143B, and U2OS), a normal osteoblast cell line (hFOB1.19), and a mouse osteosarcoma cell line (K7M2) were purchased from the Cell Bank of China Academy of Sciences (Shanghai, China) Cell lines were periodically authenticated via STR profiling. MNNG/HOS, MG-63, and U2OS cells were cultured in α-MEM (HyClone, USA) supplemented with 10% fetal bovine serum (Gibco, USA). The 143B cells were cultured in McCoy’s 5A medium (Gibco, USA) supplemented with 10% fetal bovine serum. The hFOB1.19 cells were maintained in Dulbecco’s modified Eagle’s medium (DMEM)/F-12 (Gibco, USA) medium with 10% fetal bovine serum. K7M2-WT cells were cultured in DMEM, high glucose (Gibco, USA) supplemented with 10% fetal bovine serum. All the cell lines were maintained at 37 °C with 5% CO_2_ in a humidified incubator. Canagliflozin was purchased from SELLECK (S2760, USA). CHX (#HY-12320, Med Chem Express) was used at 1 μM.

### Syngeneic tumor model treatment protocol

All animal procedures were performed in a specific pathogen free situation according to the guidelines of the ethics committee of Tongji Medical College, Huazhong University of Science and Technology (Wuhan, China). C57BL/6 mice (6–8 weeks, male) were purchased from Vitalriver (Beijing, China). The animal experimental procedures were approved by the Ethics Committee of Tongji Medical College (HUST, Wuhan, China). K7M2 cells (5 × 10^6^ in 100 μl 1 × PBS) were injected s.c. into the right flank of the mice and the mice were randomly divided into four groups (*n* = 5). The volume of the grafts was measured every other day. Tumor volumes were calculated using the following formula: tumor volume (mm^3^) = (L × W^2^) ÷ 2. Mice were sacrificed on day 21 or when tumor volume reached 1000 mm^3^.The mass of the grafts was calculated from standard measurements. Treatment with the SGLT2 inhibitor canagliflozin at doses of 30 mg/kg daily was given by the oral administration at the same time as the transplantation of tumor. At 5 and 10 days after implantation, 500 μg/kg of 2’3’-cGAMP was intraperitoneal injected to the mice.

### Statistical analysis

Data are expressed as mean ± SD of at least three independent experiments. GraphPad Prism 6 software (GradPad Software, Inc.) was used for all statistical analyses. Statistical analyses were performed using one- or two-sided paired Student’s *t* test for single comparison and one- or two-way ANOVA with a post hoc test for multiple comparisons, and the results were considered significant at *p* < 0.05.

Other methods are provided in Supplementary data. The original western blots are provided in Original WB blots.

## Supplementary information

Supplementary data

Original WB blots

Reproducibility checklist

## Data Availability

Please contact the corresponding author ZZ (zhicaizhang@126.com) for data requests.

## References

[1] Isakoff MS, Bielack SS, Meltzer P, Gorlick R (2015). Osteosarcoma: current treatment and a collaborative pathway to success. J Clin Oncol.

[2] Bernthal NM, Federman N, Eilber FR, Nelson SD, Eckardt JJ, Eilber FC (2012). Long-term results (>25 years) of a randomized, prospective clinical trial evaluating chemotherapy in patients with high-grade, operable osteosarcoma. Cancer.

[3] Link MP, Goorin AM, Miser AW, Green AA, Pratt CB, Belasco JB (1986). The effect of adjuvant chemotherapy on relapse-free survival in patients with osteosarcoma of the extremity. N Engl J Med.

[4] Collins M, Wilhelm M, Conyers R, Herschtal A, Whelan J, Bielack S (2013). Benefits and adverse events in younger versus older patients receiving neoadjuvant chemotherapy for osteosarcoma: findings from a meta-analysis. J Clin Oncol.

[5] Meyers PA, Healey JH, Chou AJ, Wexler LH, Merola PR, Morris CD (2011). Addition of pamidronate to chemotherapy for the treatment of osteosarcoma. Cancer.

[6] Kansara M, Teng MW, Smyth MJ, Thomas DM (2014). Translational biology of osteosarcoma. Nat Rev Cancer.

[7] Rieg T, Vallon V (2018). Development of SGLT1 and SGLT2 inhibitors. Diabetologia.

[8] Vallon V, Thomson SC (2017). Targeting renal glucose reabsorption to treat hyperglycaemia: the pleiotropic effects of SGLT2 inhibition. Diabetologia.

[9] Scafoglio C, Hirayama BA, Kepe V, Liu J, Ghezzi C, Satyamurthy N (2015). Functional expression of sodium-glucose transporters in cancer. Proc Natl Acad Sci USA.

[10] Koepsell H (2017). The Na(+)-D-glucose cotransporters SGLT1 and SGLT2 are targets for the treatment of diabetes and cancer. Pharm Ther.

[11] Taylor SI, Blau JE, Rother KI (2015). SGLT2 inhibitors may predispose to ketoacidosis. J Clin Endocrinol Metab.

[12] Zhou J, Zhu J, Yu SJ, Ma HL, Chen J, Ding XF (2020). Sodium-glucose co-transporter-2 (SGLT-2) inhibition reduces glucose uptake to induce breast cancer cell growth arrest through AMPK/mTOR pathway. Biomed Pharmacother.

[13] Xie Z, Wang F, Lin L, Duan S, Liu X, Li X (2020). An SGLT2 inhibitor modulates SHH expression by activating AMPK to inhibit the migration and induce the apoptosis of cervical carcinoma cells. Cancer Lett.

[14] Shiba K, Tsuchiya K, Komiya C, Miyachi Y, Mori K, Shimazu N (2018). Canagliflozin, an SGLT2 inhibitor, attenuates the development of hepatocellular carcinoma in a mouse model of human NASH. Sci Rep.

[15] Villani LA, Smith BK, Marcinko K, Ford RJ, Broadfield LA, Green AE (2016). The diabetes medication Canagliflozin reduces cancer cell proliferation by inhibiting mitochondrial complex-I supported respiration. Mol Metab.

[16] Kaji K, Nishimura N, Seki K, Sato S, Saikawa S, Nakanishi K (2018). Sodium glucose cotransporter 2 inhibitor canagliflozin attenuates liver cancer cell growth and angiogenic activity by inhibiting glucose uptake. Int J Cancer.

[17] Zhou G, Wu H, Lin J, Lin R, Feng B, Liu Z. TRIM21 is decreased in colitis-associated cancer and negatively regulates epithelial carcinogenesis. Inflamm Bowel Dis. 2020;27:458–468. 10.1093/ibd/izaa229 32860065

[18] Si W, Zhou J, Zhao Y, Zheng J, Cui L (2020). SET7/9 promotes multiple malignant processes in breast cancer development via RUNX2 activation and is negatively regulated by TRIM21. Cell Death Dis.

[19] Li Y, Shi Z, Chen L, Zheng S, Li S, Xu B (2017). Discovery of a potent, selective renal sodium-dependent glucose cotransporter 2 (SGLT2) inhibitor (HSK0935) for the treatment of type 2 diabetes. J Med Chem.

[20] Ohtake Y, Sato T, Matsuoka H, Kobayashi T, Nishimoto M, Taka N (2012). C-Aryl 5a-carba-beta-d-glucopyranosides as novel sodium glucose cotransporter 2 (SGLT2) inhibitors for the treatment of type 2 diabetes. Bioorg Med Chem.

[21] Pantelidou C, Sonzogni O, De Oliveria Taveira M, Mehta AK, Kothari A, Wang D (2019). PARP inhibitor efficacy depends on CD8(+) T-cell recruitment via intratumoral STING pathway activation in BRCA-deficient models of triple-negative breast cancer. Cancer Disco.

[22] Wu J, Sun L, Chen X, Du F, Shi H, Chen C (2013). Cyclic GMP-AMP is an endogenous second messenger in innate immune signaling by cytosolic DNA. Science.

[23] Chandra D, Quispe-Tintaya W, Jahangir A, Asafu-Adjei D, Ramos I, Sintim HO (2014). STING ligand c-di-GMP improves cancer vaccination against metastatic breast cancer. Cancer Immunol Res.

[24] Škrnjug I, Guzmán CA, Ruecker C (2014). Cyclic GMP-AMP displays mucosal adjuvant activity in mice. PLoS ONE.

[25] Lai J, Fu Y, Tian S, Huang S, Luo X, Lin L (2021). Zebularine elevates STING expression and enhances cGAMP cancer immunotherapy in mice. Mol Ther.

[26] Wu S, Zhang Q, Zhang F, Meng F, Liu S, Zhou R (2019). HER2 recruits AKT1 to disrupt STING signalling and suppress antiviral defence and antitumour immunity. Nat Cell Biol.

[27] Zhang X, Zhang X, Liu X, Qi P, Wang H, Ma Z (2019). MicroRNA-296, a suppressor non-coding RNA, downregulates SGLT2 expression in lung cancer. Int J Oncol.

[28] Komatsu S, Nomiyama T, Numata T, Kawanami T, Hamaguchi Y, Iwaya C (2020). SGLT2 inhibitor ipragliflozin attenuates breast cancer cell proliferation. Endocr J.

[29] Odell ID, Flavell RA (2019). HER2 joins AKT to inhibit STING immunity. Nat Cell Biol.

[30] Abe T, Barber GN (2014). Cytosolic-DNA-mediated, STING-dependent proinflammatory gene induction necessitates canonical NF-kappaB activation through TBK1. J Virol.

[31] Balka KR, Louis C, Saunders TL, Smith AM, Calleja DJ, D’Silva DB (2020). TBK1 and IKKepsilon act redundantly to mediate STING-induced NF-kappaB responses in myeloid cells. Cell Rep.

[32] Yum S, Li M, Fang Y, Chen ZJ. TBK1 recruitment to STING activates both IRF3 and NF-kappaB that mediate immune defense against tumors and viral infections. Proc Natl Acad Sci USA. 2021;118:e2100225118. 10.1073/pnas.2100225118 PMC8040795 33785602

[33] Bohnert V, Ritchie C, Li L (2020). IFN-independent STING signaling: friend or foe?. Immunity.

[34] Yamashiro LH, Wilson SC, Morrison HM, Karalis V, Chung JJ, Chen KJ (2020). Interferon-independent STING signaling promotes resistance to HSV-1 in vivo. Nat Commun.

[35] Jing W, McAllister D, Vonderhaar EP, Palen K, Riese MJ, Gershan J (2019). STING agonist inflames the pancreatic cancer immune microenvironment and reduces tumor burden in mouse models. J Immunother Cancer.

[36] Tumeh PC, Harview CL, Yearley JH, Shintaku IP, Taylor EJ, Robert L (2014). PD-1 blockade induces responses by inhibiting adaptive immune resistance. Nature.

[37] Bertucci F, Goncalves A (2017). Immunotherapy in breast cancer: the emerging role of PD-1 and PD-L1. Curr Oncol Rep.

[38] Feng M, Xiong G, Cao Z, Yang G, Zheng S, Song X (2017). PD-1/PD-L1 and immunotherapy for pancreatic cancer. Cancer Lett.

[39] Gu L, Chen M, Guo D, Zhu H, Zhang W, Pan J (2017). PD-L1 and gastric cancer prognosis: a systematic review and meta-analysis. PLoS ONE.

[40] Mocan T, Sparchez Z, Craciun R, Bora CN, Leucuta DC (2019). Programmed cell death protein-1 (PD-1)/programmed death-ligand-1 (PD-L1) axis in hepatocellular carcinoma: prognostic and therapeutic perspectives. Clin Transl Oncol.

[41] Boussiotis VA (2016). Molecular and biochemical aspects of the PD-1 checkpoint pathway. N Engl J Med.

[42] Sharma P, Allison JP (2015). The future of immune checkpoint therapy. Science.

[43] Fu J, Kanne DB, Leong M, Glickman LH, McWhirter SM, Lemmens E (2015). STING agonist formulated cancer vaccines can cure established tumors resistant to PD-1 blockade. Sci Transl Med.

[44] Moore E, Clavijo PE, Davis R, Cash H, Van Waes C, Kim Y (2016). Established T cell-inflamed tumors rejected after adaptive resistance was reversed by combination STING activation and PD-1 pathway blockade. Cancer Immunol Res.

[45] Ding Q, He D, He K, Zhang Q, Tang M, Dai J (2015). Downregulation of TRIM21 contributes to hepatocellular carcinoma carcinogenesis and indicates poor prognosis of cancers. Tumour Biol.

[46] Jin Y, Zhao X, Zhang Q, Zhang Y, Fu X, Hu X (2020). Cancer-associated mutation abolishes the impact of TRIM21 on the invasion of breast cancer cells. Int J Biol Macromol.

[47] Pan JA, Sun Y, Jiang YP, Bott AJ, Jaber N, Dou Z (2016). TRIM21 ubiquitylates SQSTM1/p62 and suppresses protein sequestration to regulate redox homeostasis. Mol Cell.

[48] Foss S, Watkinson RE, Grevys A, McAdam MB, Bern M, Hoydahl LS (2016). TRIM21 immune signaling is more sensitive to antibody affinity than its neutralization activity. J Immunol.

[49] Zhang Z, Bao M, Lu N, Weng L, Yuan B, Liu YJ (2013). The E3 ubiquitin ligase TRIM21 negatively regulates the innate immune response to intracellular double-stranded DNA. Nat Immunol.

[50] Li D, Wu R, Guo W, Xie L, Qiao Z, Chen S (2019). STING-mediated IFI16 degradation negatively controls type I interferon production. Cell Rep.

[51] Zhou W, Zhang Y, Zhong C, Hu J, Hu H, Zhou D (2018). Decreased expression of TRIM21 indicates unfavorable outcome and promotes cell growth in breast cancer. Cancer Manag Res.

[52] Zhou G, Wu H, Lin J, Lin R, Feng B, Liu Z (2021). TRIM21 is decreased in colitis-associated cancer and negatively regulates epithelial carcinogenesis. Inflamm Bowel Dis.

