# Supplementary material for: SGLT2 inhibitor activates the STING/IRF3/IFN-β pathway and induces immune infiltration in osteosarcoma
Source: Cell Death Dis. 2022 Jun 3;13(6):523. doi: 10.1038/s41419-022-04980-w (PMC9166744; doi:10.1038/s41419-022-04980-w)
Supplement: Supplementary file 1 — Supplementary data [file 41419_2022_4980_MOESM1_ESM.docx]

**Supplementary Data**

**Supplementary methods and materials.**

**Western blot of cells and tissues**

The rationality of using human tissue (8 pairs of matched osteosarcoma/adjacent non-tumor tissues, supplementary table 4) was audited by the Local Ethics Committee (Tongji Medical College, China) ([2020] IEC-J (202)). All patients or their guardians provided written informed consent. Tumor tissues (after grinding) Cells were lysed on ice in RIPA buffer (Beyotime Institute of Biotechnology, Guangzhou, China) containing a protease inhibitor cocktail (Beyotime Institute of Biotechnology, Guangzhou, China). The protein content in the lysates was determined using a BCA protein assay kit (Beyotime Biotechnology, China). Equal amounts of total protein from different samples were separated by SDS-PAGE gels at 100 V for 1.5 h and transferred to 0.45 μm polyvinylidene difluoride (PVDF) membranes (Pierce Biotechnology, USA) at 300 mA for 1 h. Then, the membranes were blocked with 5% skim milk powder in Tris buffered saline Tween（TBST）for 1 h at room temperature and treated with specific primary antibodies overnight at 4 °C. On the next day, the membranes were washed with TBST and incubated with an HRP-conjugated secondary antibody (BOSTER, China). Finally, the membranes were treated with ECL detection reagents, exposed using the ChemiDoc XRS imaging system (Bio‑Rad Laboratories, Inc. Hercules, CA, USA), and analyzed with the Image Lab software (Bio‑Rad Laboratories, Inc. Hercules, CA, USA). GAPDH was used to ensure equal protein loading. STING antibody (19851-1-AP, 1:1000), TRIM21 antibody (12108-1-AP, 1:1000), GAPDH antibody (10494-1-AP, 1:3000), AKT antibody (10176-2-AP, 1:1000), and Phospho-AKT antibody (66444-1-Ig, 1:1000) were purchased from Proteintech. SGLT2 antibody (14210, 1:1000), IRF3 antibody (4302, 1:1000), and Phospho-IRF3 antibody (37829S,1:1000) were purchased from Cell Signaling Technology. Immunoreactivity was analyzed by Image Lab software. Protein expression was normalized to GAPDH.

**ELISA assay**

Cells were lysed and the supernatant was collected after centrifugation. Standard and samples were added to wells pre-incubated with IFN-β monoclonal antibody. After incubation for 2 h, diluted detection antibody was added, and the cells were incubated for 1 h. Then, diluted HRP conjugate was added, followed by incubation for 30 min and addition of chromogenic substrate. After development in the dark for 30 min, stop solution was added, and the plate was transferred to the enzyme microplate reader to detect the absorbance (OD) at 450 nm. Then, we used a curve-fitting statistical software to fit the standards with a four-parameter logistic curve and calculate the results for the test samples.

**Immunohistochemistry (IHC)**

The tissue microarray slides were purchased from Bioaitech ([L1024901](http://www.bioaitech.com/chip-design/a72912c4a8ee4bf183ff534750c0e6f4.html) and [L714901](http://www.bioaitech.com/chip-design/43dedfaeb84e4f76813494ec00e8807a.html)). IHC analysis was performed to determine the protein expression level with the SGLT2 antibody (Proteintech, 24654-1-AP, 1:2000). Two independent pathologists, who were uninformed with the patient data and histopathological features of the samples, were responsible for reviewing and scoring the degree of immunostaining separately. Staining intensity was graded/scored in a blinded fashion: 1 = weak staining at ×100 magnification but little or no staining at ×40 magnification; 2 = medium staining at ×40 magnification; 3 = strong staining at ×40 magnification. A final staining index was calculated using the formula: staining intensity × percentage.

**Immunofluorescence Staining**

Briefly, fresh tumor tissues were deparaffinized and subjected to antigen retrieval using EDTA buffer (#93283, Sigma-Aldrich) pH = 8.0 and boiled for 20minutes at 97° C in a pressure-boiling container. Slides were then incubated with dual endogenous peroxidase block for 10 minutes at room temperature and subsequently with a blocking solution containing 0.3% bovine serum albumin in 0.05% Tween solution for 30 minutes. Tumor sections were incubated with indicated primary antibody for CD3, CD4, and CD8 at 4°C overnight. After washed three times with PBS, sections were incubated with secondary antibody that was conjugated with Alexa Fluor 488 dye or Alexa Fluor 594 dye (Thermo Fisher Scientific) for 1 hr at room temperature. Nuclei were highlighted using 4’,6-Diamidino-2-Phenylindole (DAPI). Images were captured using Zeiss laser confocal microscope (LSM780).

**Immunoprecipitation**

Cell lysis solution was incubated with an appropriate amount of antibody at 4 °C for 3 h, followed by incubation with protein A agarose (Vigorous Biotechnology, Beijing, China) for 1 h.The immune precipitates were washed three times using a lysis buffer solution, followed by elution with SDS loading buffer. The eluent was subjected to Western blot analysis.

**Liquid chromatography-tandem mass spectrometry/mass spectrometry** **analysis**

The 293T cells transfected with an SGLT2-expressing plasmid were used to identify novel SGLT2-binding proteins. The IgG antibody and protein A+G agarose (#P2012, Beyotime Institute of Biotechnology, Guangzhou, China) were added to the cell lysates and incubated for 30mins and washed to remove non-specific binding proteins. SGLT2 was immunoprecipitated using an anti-SGLT2 antibody or isotype IgG antibody and protein A+G agarose at 4 °C. Liquid chromatography-tandem mass spectrometry/mass spectrometry (LC-MS/MS) analysis was performed using a Thermo Ultimate 3000 liquid-phase column combined with a Q-Exactive Plus high-resolution mass spectrometer (Shanghai Applied Protein Technology). The data were retrieved using the software maxquant (v1.6.6) and the algorithm Andromeda, and the data were obtained from the human proteome reference database of UniProt. Proteins and peptides with a false discovery rate (FDR) of 1% were selected.

**RNA sequencing**

A total of 1 µg of RNA per sample was used as the starting material for RNA sequencing (RNA-seq). RNA integrity was assessed using the RNA Nano 6000 Assay Kit of the Bioanalyzer 2100 system (Agilent Technologies, CA, USA). Clean data (clean reads) were obtained by removing reads containing adapter, reads containing ploy-N and low quality reads from raw data. At the same time, Q20, Q30 and GC content the clean data were calculated. All the downstream analyses were based on the clean data with high quality. Sequencing libraries were generated using the NEBNext Ultra RNA Library Prep Kit for Illumina (NEB, USA) following the manufacturer’s instructions, and index codes were added to attribute sequences to each sample. Clustering of the samples was performed on the cBot Cluster Generation System using the TruSeq PE Cluster Kit v3-cBot-HS (Illumina) according to the manufacturer’s instructions. After cluster generation, libraries were sequenced on an Illumina Novaseq platform, and 150-bp paired-end reads were generated. FeatureCounts v1.5.0-p3 was used to count the read numbers mapped to each gene. Differential expression analysis (two biological replicates per condition) was performed using the DESeq2 R package (1.16.1), and the clusterProfiler R package was used to test the statistical enrichment of differentially expressed genes (DEGs) in KEGG (Kyoto Encyclopedia of Genes and Genomes) pathways. Three replicates were performed in each group.

**Quantitative real-time PCR**

Total RNA was extracted from the cells using the TRIzol reagent (Invitrogen, USA). First-strand cDNA was generated using the random hexamer primer provided in the first-strand cDNA synthesis kit (PrimeScript™ RT reagent Kit, Code No. RR037A). Then, quantitative real-time PCR analysis (qRT-PCR) was conducted using a PCR kit (TB Green™ Fast qPCR Mix, Code No. RR430A) according to the manufacturer’s protocols. Specific primers for each gene (Supplementary table 1) were designed using the Primerbank database. All the experiments were performed in triplicate and calibrated to GAPDH, and we used the 2^-ΔCt^ method to quantify the fold change.

**RNA interference**

The shRNAs were procured from Sigma-Aldrich. Lipofectamine 3000 (Invitrogen, USA) and Opti-MEM medium (Invitrogen, USA) were used for the transfection reactions; Lipofectamine 3000 was used to transfect 293 T cells to shRNA plasmids and viral packaging plasmids (pVSV-G and pEXQV). At 24 h after transfection, the medium was replaced with fresh DMEM containing 10% FBS and 1 mM sodium pyruvate, and 48 h post-transfection, the virus culture medium was collected and added to the MNNG/HOS and MG-63 cells supplemented with 12 μg/mL of polybrene. At 24 h after infection, the infected cells were selected with 10 μg/mL of puromycin. The shRNA sequences are provided in the Supplementary table 2.

**Bioinformatic data mining**

The GEPIA web tool was used to determine the SGLT2 expression level in cancers. The TIMER (Tumor IMmune Estimation Resource; <http://timer.cistrome.org/>) web tool was used to predict the correlation between the expression level of SGLT2 and the infiltration level of immune cells in osteosarcoma.

**Flow cytometry analysis**

For flow cytometry analysis of the mouse tissue samples, single-cell suspensions were prepared and stained with the following antibodies: APC-conjugated CD45 antibody (Biolegend, 103,112, USA); FITC conjugated CD4 antibody (Biolegend, 100,510, USA); PE-conjugated CD8 antibody (Biolegend, 100,708, USA) . After 15 min incubation at room temperature, cells were washed three times for 10 min each time with PBS, the cells were then resuspended in PBS and analyzed by flow cytometry. Data was analyzed with the software FlowJo.

**Supplementary Figure 1. Supplementary data to Figure 2.**


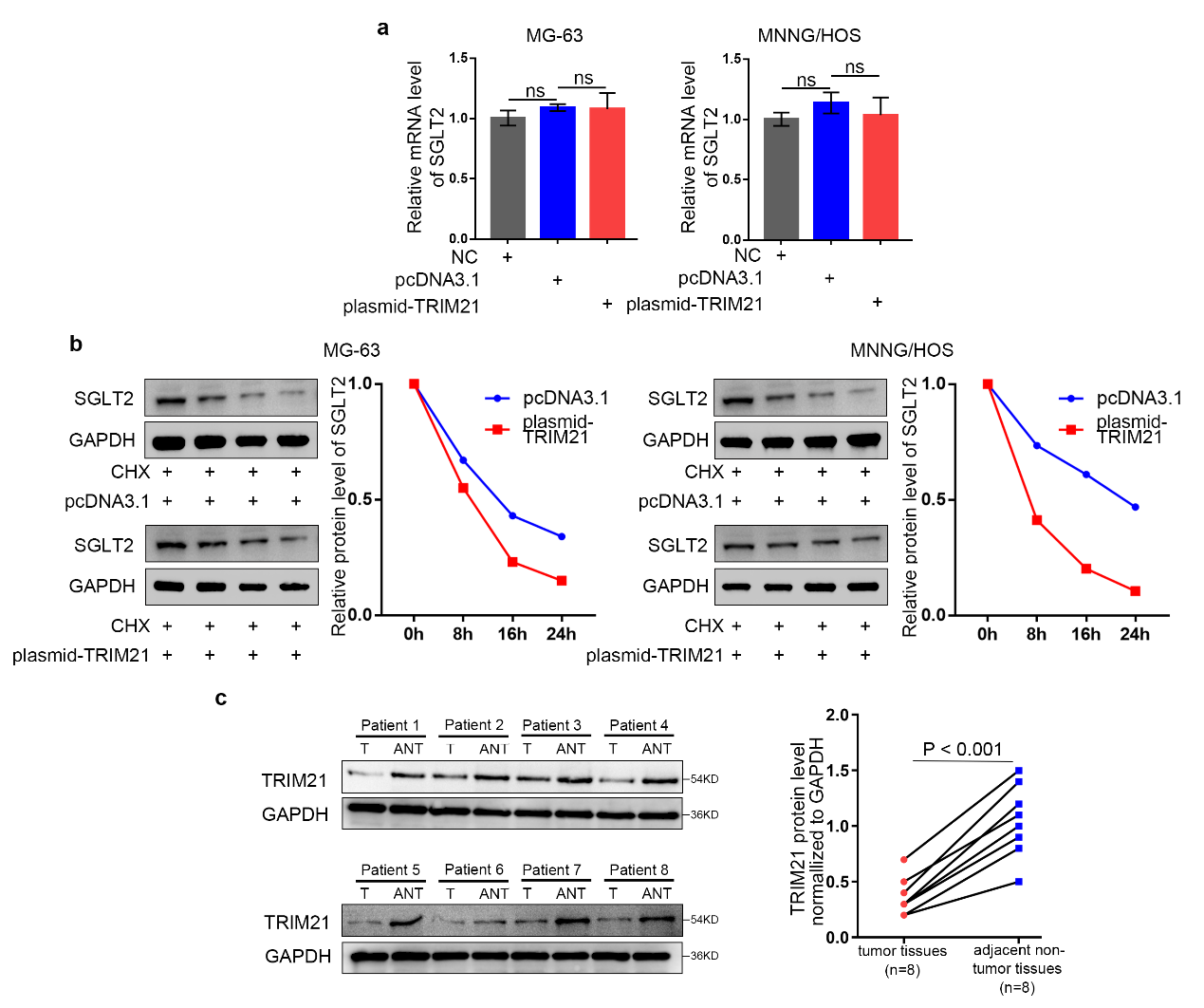


**a.** RT-PCR analysis of the mRNA expression levels in MG-63 and MNNG/HOS cell lines infected with or without pcDNA3.1 or TRIM21 plasmids for 48 h. Data are presented as the mean ± SD of three independent experiments (ns, not significant).

**b.** Western Blot to show the SGLT2 expression in MG-63 and MNNG/HOS cells. The cells were treated with cycloheximide for different duration.

**c.** Western blot analysis of the protein expression of SGLT2 in 8 paired osteosarcoma tumor tissues (T) and the matched adjacent normal tissues (ANT) of the same patient. GAPDH served as an internal reference.

**Supplementary Figure 2. Supplementary data to Figure 3.**


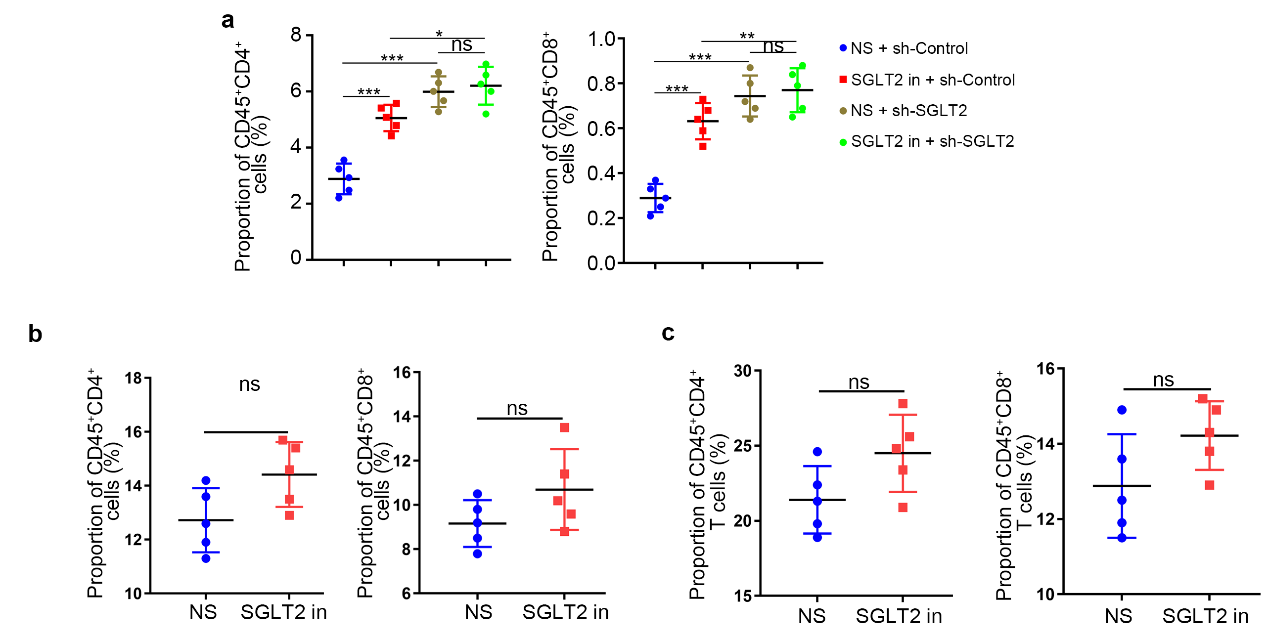


1. Flow cytometry analysis of the percentage of infiltrated CD45^+^CD4^+^ and CD45^+^CD8^+^ T cells in the K7M2 tumors. Data are presented as the mean ± SD of five independent experiments (***, P < 0.001; **, P < 0.01; *, P < 0.05; ns, not significant).
2. Proportions of CD45^+^CD4^+^ and CD45^+^CD8^+^ cells in blood collected from the mice. Data are presented as the mean ± SD of five independent experiments (ns, not significant).
3. Proportions of CD45^+^CD4^+^ and CD45^+^CD8^+^ cells in spleen collected from the mice. Data are presented as the mean ± SD of five independent experiments (ns, not significant).

**Supplementary Figure 3. Supplementary data to Figure 4.**


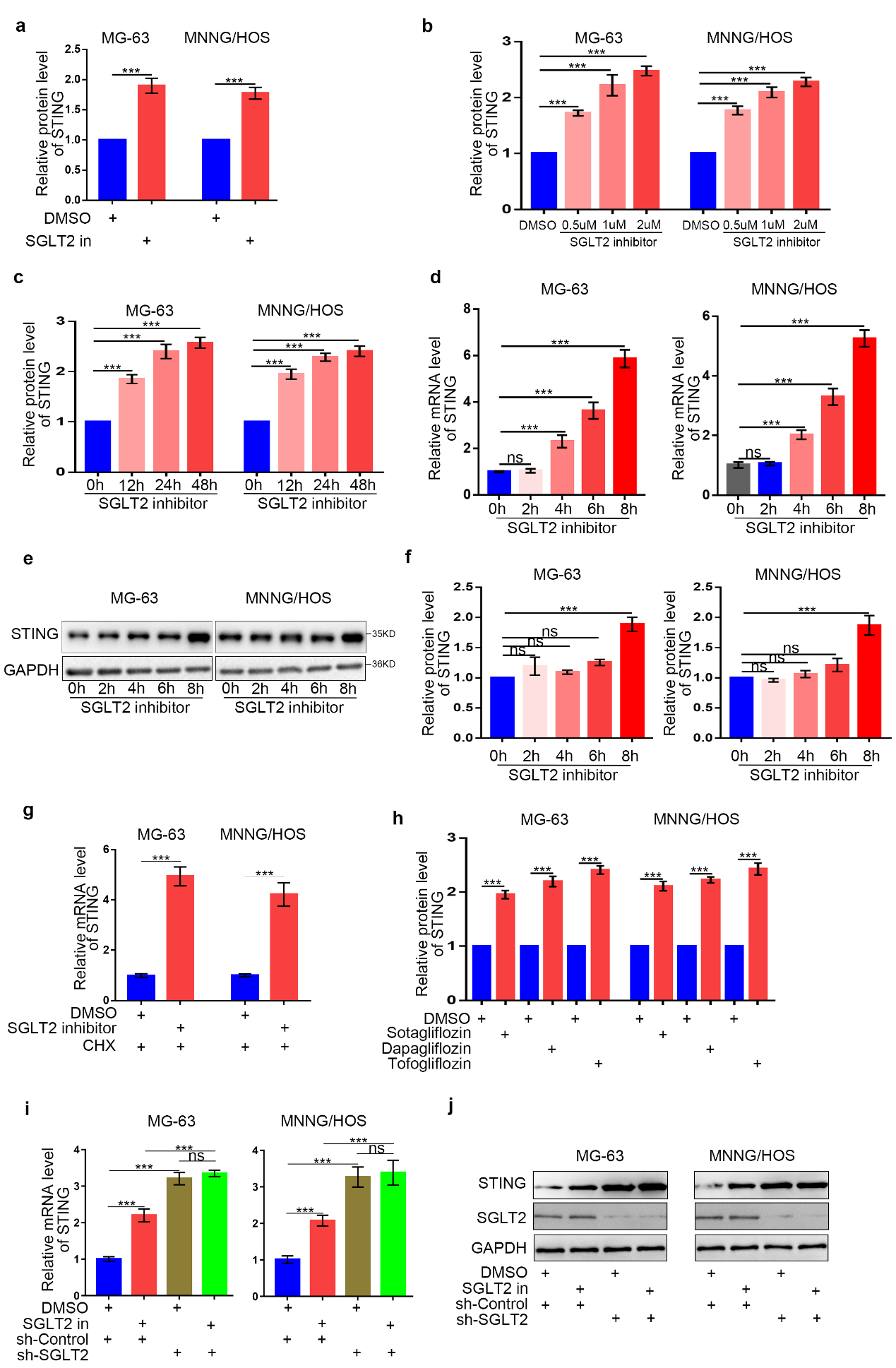


**a.** The relative protein level of STING in Figure 3c (n= 3). GAPDH served as an internal reference.

**b.** The relative protein level of STING in Figure 3e (n= 3). GAPDH served as an internal reference.

**c.** The relative protein level of STING in Figure 3g (n= 3). GAPDH served as an internal reference.

**d.** RT-PCR analyses of the mRNA expression levels of STING in MG-63 and MNNG/HOS cell lines treated with SGLT2 inhibitor (canagliflozin, 1 uM) for 0, 2, 4, 6, or 8 h. Data are presented as the mean ± SD of three independent experiments (*, P < 0.05; **, P < 0.01; ***, P < 0.001).

**e.** Western blot analyses of the protein expression levels of STING in MG-63 and MNNG/HOS cell lines treated with SGLT2 inhibitor (canagliflozin, 1 uM) for 0, 2, 4, 6, or 8 h.

**f.** The relative protein level of STING in Supplementary figure 3e (n= 3). GAPDH served as an internal reference.

**g.** RT-PCR analyses of the mRNA expression levels of STING in MG-63 and MNNG/HOS cell lines treated with or without SGLT2 inhibitor (canagliflozin, 1 uM). Cells were treated with CHX for 8 h before harvesting. Data are presented as the mean ± SD of three independent experiments (*, P < 0.05; **, P < 0.01; ***, P < 0.001).

**h.** The relative protein level of STING in Figure 3i (n= 3). GAPDH served as an internal reference.

**i and j.** RT-PCR (i) and Western Blot (j) analyses of the mRNA and protein expression levels of STING in MG-63 and MNNG/HOS cell lines treated with SGLT2 inhibitor (canagliflozin, 1 uM) and/or infected with sh-SGLT2. GAPDH served as an internal reference. Data are presented as the mean ± SD of three independent experiments (*, P < 0.05; **, P < 0.01; ***, P < 0.001).

**Supplementary Figure 4. Supplementary data to Figure 6.**


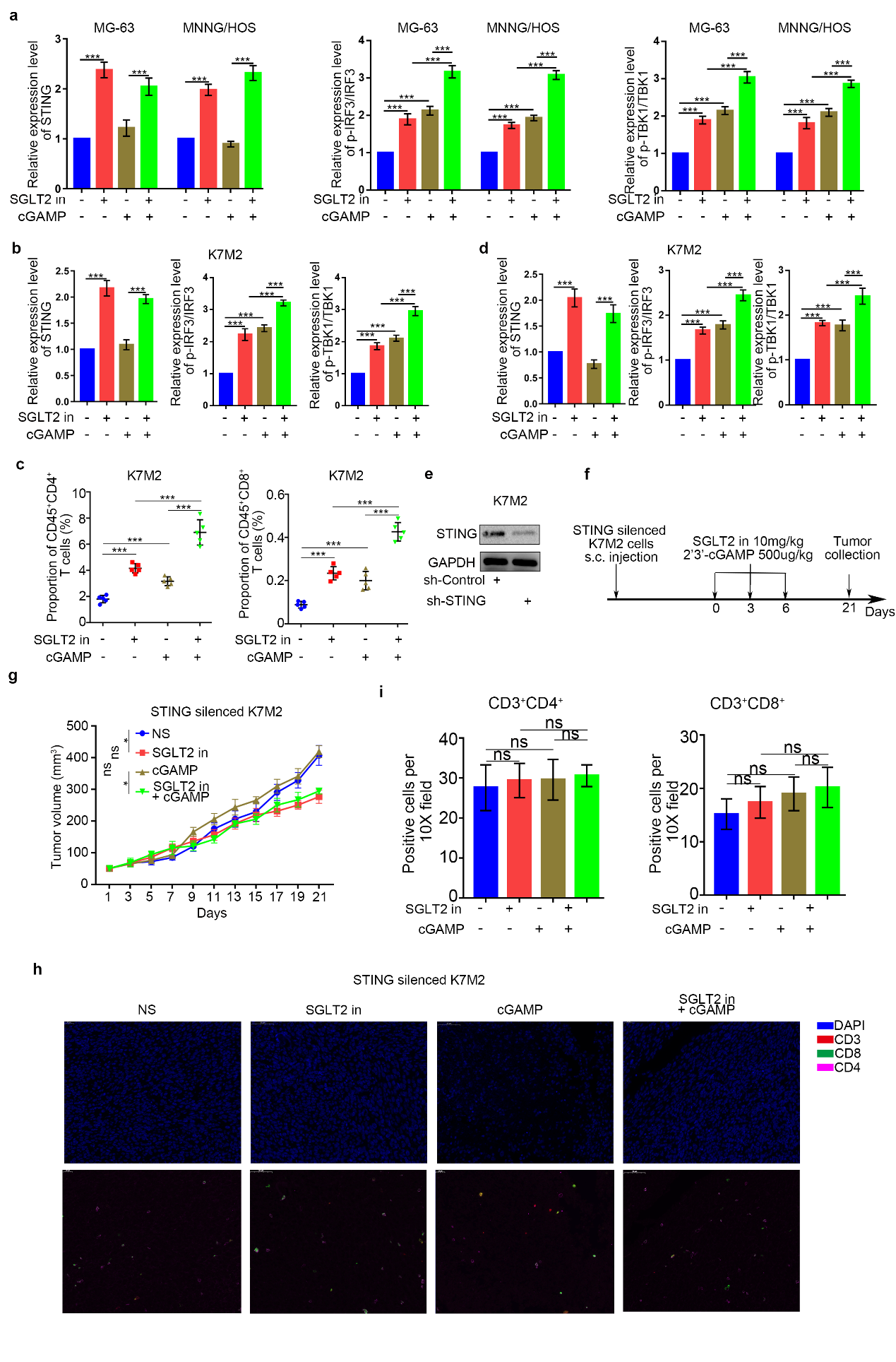


1. The relative protein levels in Figure 5c (n= 3). GAPDH served as an internal reference.
2. The relative protein levels in Figure 5e (n= 3). GAPDH served as an internal reference.
3. Flow cytometry analysis of the percentage of infiltrated CD45^+^CD4^+^ and CD45^+^CD8^+^ T cells per 10,000 cells. Data are presented as the mean ± SD of five independent experiments (***, P < 0.001).
4. The relative protein levels in Figure 5i (n= 3). GAPDH served as an internal reference.
5. Western Blot to show the STING expression in K7M2 cells. GAPDH served as an internal reference.
6. Schematic diagram of the procedure of the in vivo experiments; the doses of cGAMP and SGLT2 inhibitor (canagliflozin) are indicated above.
7. K7M2 tumor growth curves of different groups (n = 5/group; ***, P < 0.001).

h and i. Immunofluorescence analysis of the percentage of infiltrated CD3^+^CD4^+^ and CD3^+^CD8^+^ T cells per 10X field. Data are presented as the mean ± SD of five independent experiments (ns, not significant).

**Supplementary Figure 5. Supplementary data to Figure 7.**


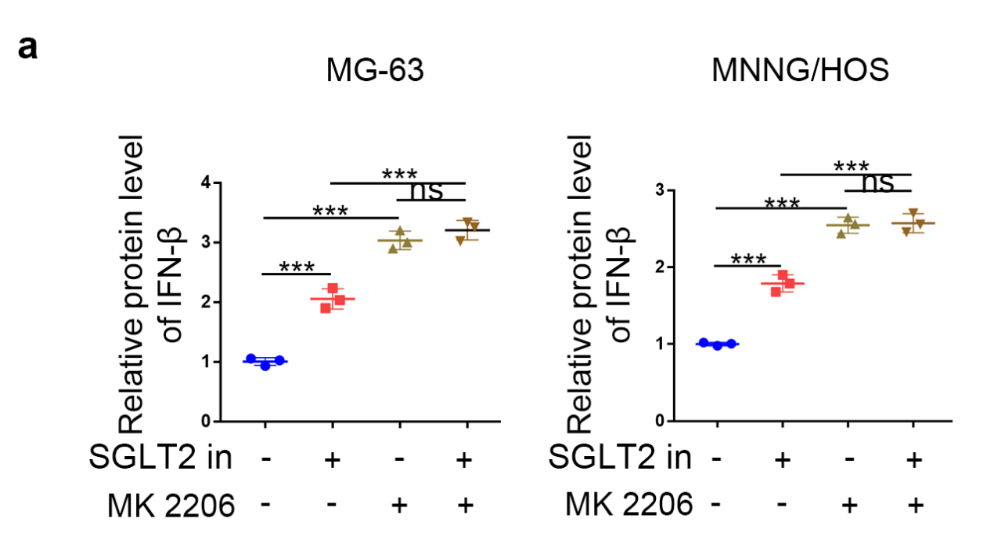


**a.** ELISA analysis of the protein expression level of IFN-β in MG-63 and MNNG/HOS cell lines treated with canagliflozin (1 uM) and/or MK 2206 (10 μM) for 48h. (**, P < 0.01; ***, P < 0.001).

**Supplementary Figure 6.** **SGLT2 inhibitor treatment enhances the anti- osteosarcoma of PD-1 antibody.**


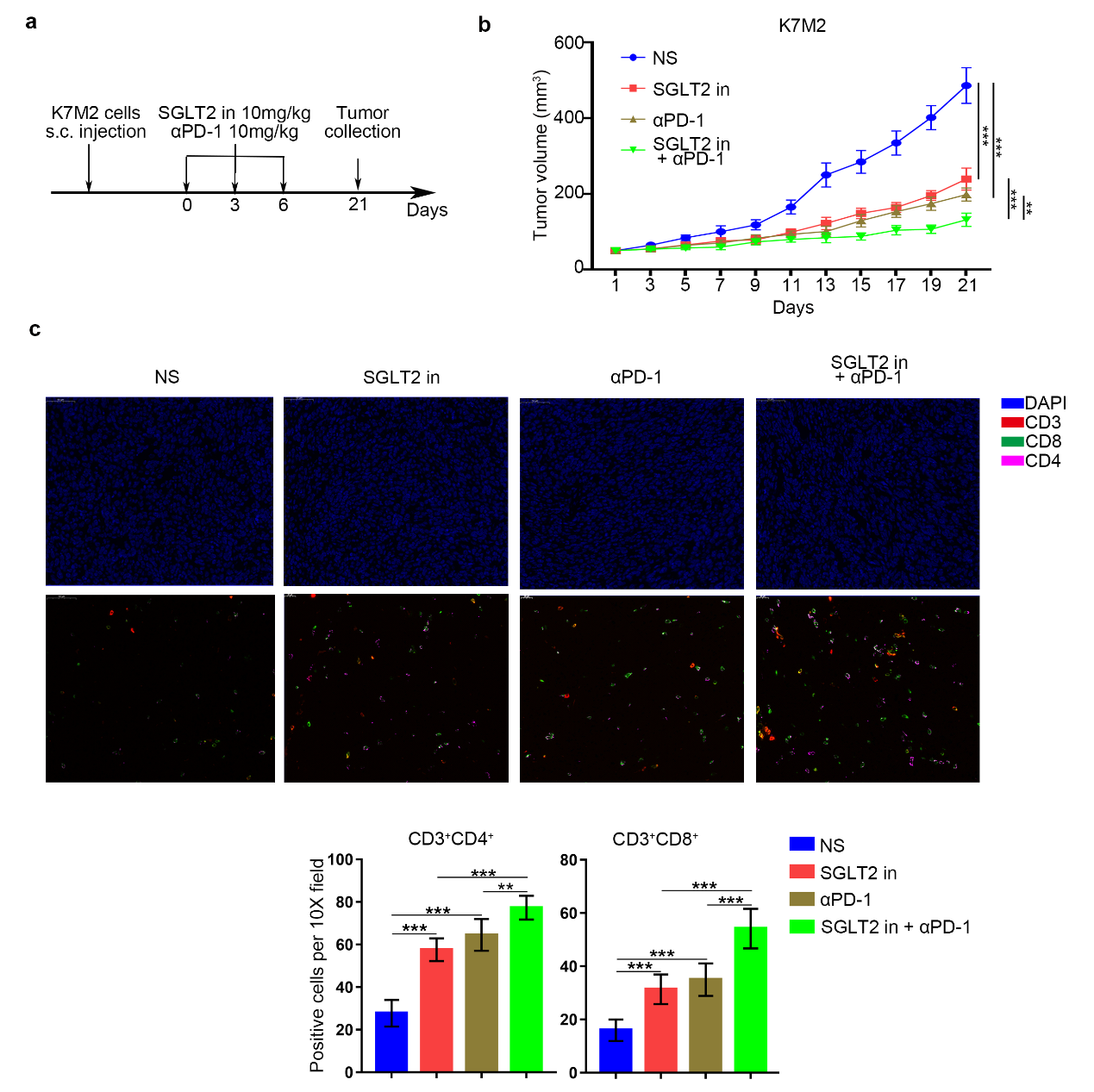


**a.** Schematic diagram of the procedure of the in vivo experiments; the doses of αPD-1 and SGLT2 inhibitor (canagliflozin) are indicated above.

**b.** K7M2 tumor growth curves of different groups (n = 5/group; ***, P < 0.001).

**c.** Immunofluorescence analysis of the percentage of infiltrated CD3^+^CD4^+^ and CD3^+^CD8^+^ T cells per 10X field. Data are presented as the mean ± SD (n = 5/group; ***, P < 0.001).

**Supplementary table 1. The primer sequences for RT-qPCR.**

| Gene | Forward primer (**5**′ - 3′) | Reverse primer (**5**′ - 3′) |
| --- | --- | --- |
| GAPDH | ATGACAATGAATACGGCTACAGCA | GCAGCGAACTTTATTGATGGTATT |
| SGLT2 | TCCTGCTGACATCCTAGTCATT | GAAGAGCGCATTCCACTCG |
| STING | CCAGAGCACACTCTCCGGTA | CGCATTTGGGAGGGAGTAGTA |
| IRF3 | AGAGGCTCGTGATGGTCAAG | AGGTCCACAGTATTCTCCAGG |
| IFNB1 | ATGACCAACAAGTGTCTCCTCC | GGAATCCAAGCAAGTTGTAGCTC |

**Supplementary table 2. The shRNA sequences.**

| Sh-TRIM21 #1 | CCGGAGGACATGTTGGGTTCATATTCTCGAGAATATGAACCCAACATGTCCTTTTTTG |
| --- | --- |
| Sh-TRIM21 #2 | CCGGTTGTCTCCTTCTACAACATAACTCGAGTTATGTTGTAGAAGGAGACAATTTTTG |
| Sh-STING #1 | CCGGGCAGTCCTATCCCATGCAAAGCTCGAGCTTTGCATGGGATAGGACTGCTTTTTG |
| Sh-STING #2 | CCGGGGTCTTTATGTGAGCCTATTGCTCGAGCAATAGGCTCACATAAAGACCTTTTTG |

**Supplementary table 3. The gene counts for common genes of cytosolic DNA-sensing pathway in the RNA sequencing data (Fig. 2a).**

| Gene name | Gene counts | | | | | | | SGLT2 in vs DMSO | |
| --- | --- | --- | --- | --- | --- | --- | --- | --- | --- |
|  | DMSO seq1 | DMSO seq2 | DMSO seq3 | SGLT2 in seq1 | | SGLT2 in seq2 | SGLT2 in seq3 | log2FoldChange | Pvalue |
| CGAS | 293 | 274 | 306 | | 251 | 261 | 262 | 0.02127 | 0.88349 |
| ISG15 | 345 | 389 | 396 | | 466 | 420 | 426 | 0.07833 | 0.59390 |
| ISG20 | 74 | 113 | 91 | | 105 | 105 | 78 | 0.22164 | 0.37655 |
| IFI6 | 383 | 382 | 378 | | 459 | 441 | 442 | 0.13427 | 0.33876 |
| IFI27L1 | 315 | 368 | 268 | | 307 | 303 | 376 | 0.12065 | 0.42960 |
| IFI27L2 | 213 | 284 | 214 | | 331 | 303 | 339 | 0.17424 | 0.38159 |
| IFI16 | 86 | 82 | 82 | | 82 | 100 | 101 | 0.05884 | 0.80357 |
| IFI27 | 28 | 30 | 24 | | 19 | 23 | 38 | 0.16748 | 0.69313 |
| IFI30 | 13 | 8 | 12 | | 7 | 12 | 13 | 0.07171 | 0.91179 |

**Supplementary table 4. The clinical characteristics of the 8 patients.**

| No. | Gender | Age | Tumor type | Position | Stage | Lung metastasis |
| --- | --- | --- | --- | --- | --- | --- |
| Patient 1 | Male | 15 | Common osteosarcoma, malignant fibrous histiocytoma like osteosarcoma | Right lower femur | IIA | N |
| Patient 2 | Male | 11 | Common osteosarcoma, chondroblastoma osteosarcoma | Right lower femur | IIA | N |
| Patient 3 | Male | 6 | Common osteosarcoma, chondroblastoma osteosarcoma | Left tibia | IIB | N |
| Patient 4 | Male | 10 | Common osteosarcoma | Lower left femur | IIA | N |
| Patient 5 | Female | 8 | Common osteosarcoma | Right femur | IIA | N |
| Patient 6 | Female | 15 | Common osteosarcoma | Left femur | IIA | N |
| Patient 7 | Female | 15 | Common osteosarcoma, osteoblast osteosarcoma | Left femur | IIB | N |
| Patient 8 | Female | 20 | Common osteosarcoma, sclerosing osteosarcoma | Right femur | IIA | N |
