# Supplementary material for: SGLT2 inhibitor activates the STING/IRF3/IFN-β pathway and induces immune infiltration in osteosarcoma
Source: Cell Death Dis. 2022 Jun 3;13(6):523. doi: 10.1038/s41419-022-04980-w (PMC9166744; doi:10.1038/s41419-022-04980-w)
Supplement: Supplementary file 2 — Original WB blots [file 41419_2022_4980_MOESM2_ESM.docx]

**Original blots**

Figure 1c

SGLT2


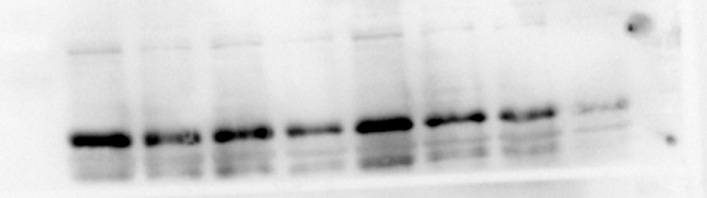




SGLT2


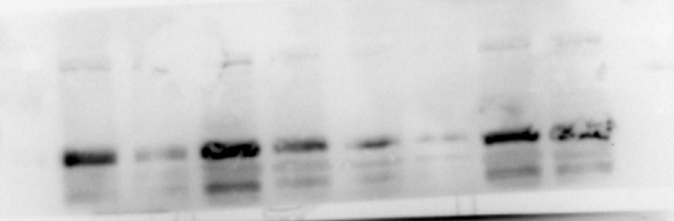





Figure 1e

SGLT2


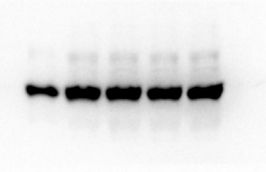


GAPDH


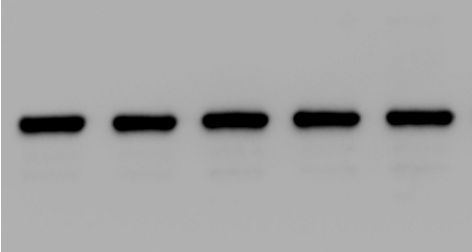


Figure 2c

SGLT2-1


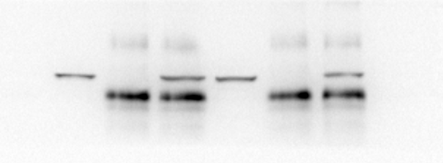


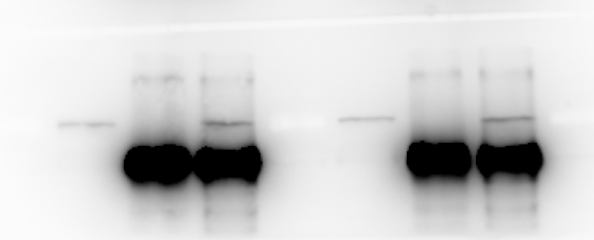


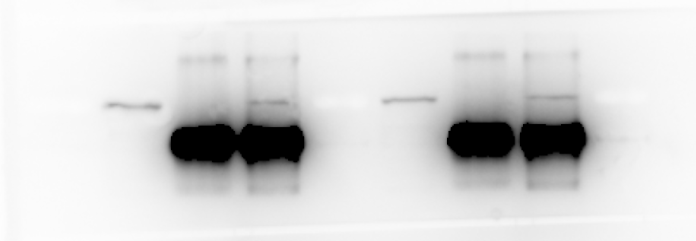


TRIM21-1


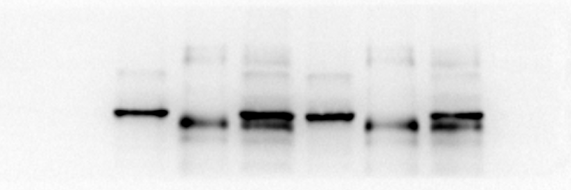


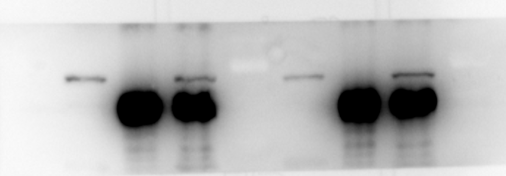


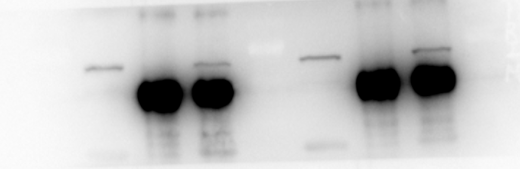


Figure 2d

TRIM21


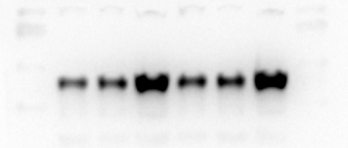


SGLT2


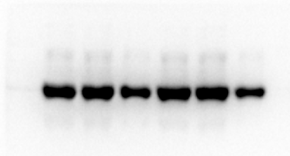


GAPDH


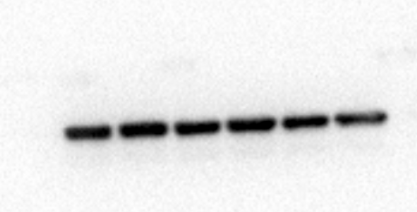


Figure 2e

SGLT2


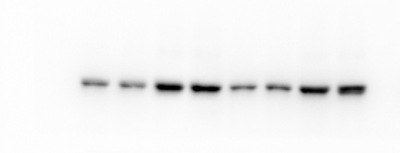


TRIM21


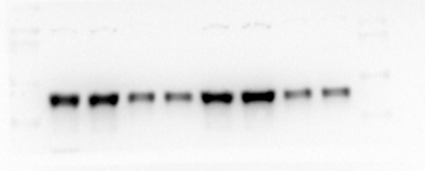


GAPDH


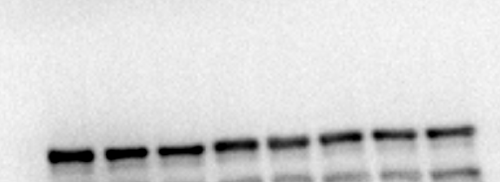


Figure 2g









Figure 2f

















Figure 2g









Figure 4c

STING


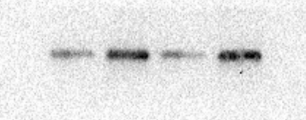


GAPDH


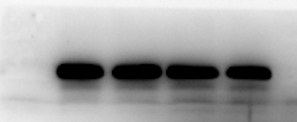


Figure 4e

STING


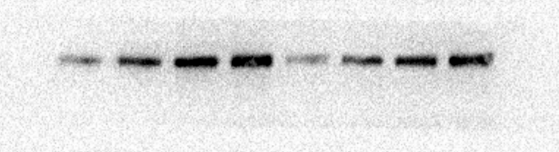


GAPDH


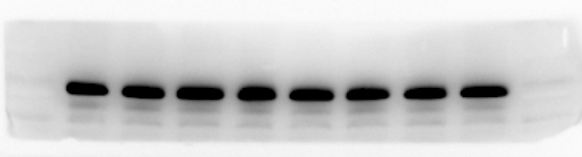


Figure 4g

STING


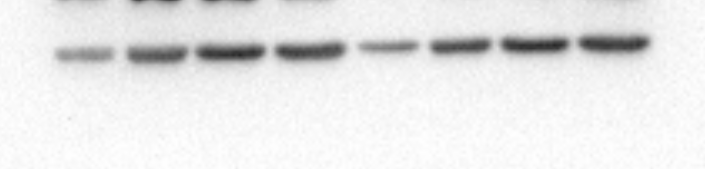


GAPDH


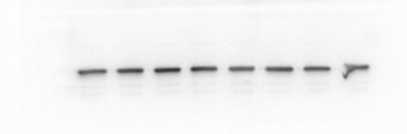


Figure 4i

STING


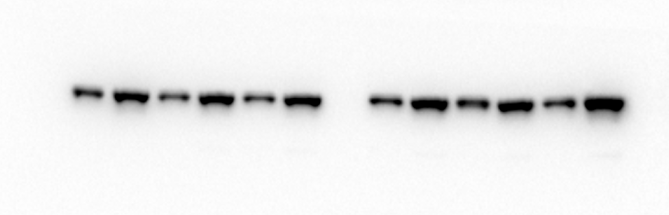


GAPDH


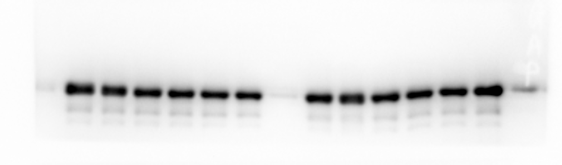


Figure 5b

p-TBK1


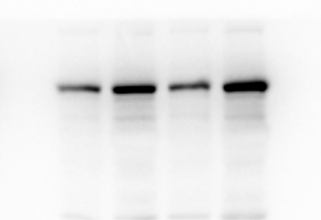


TBK1


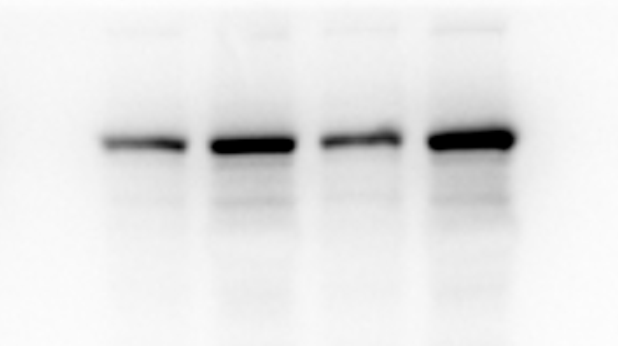


p-STING


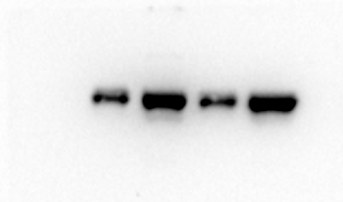











Figure 5h

p-IRF3


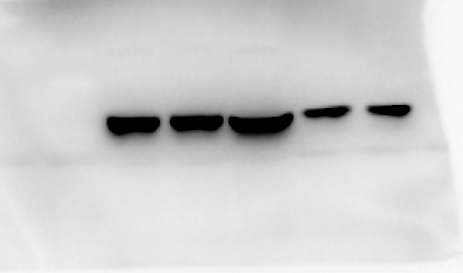


IRF3


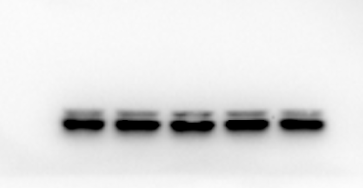


p-TBK1


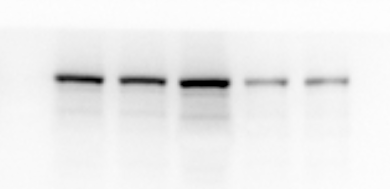


TBK1


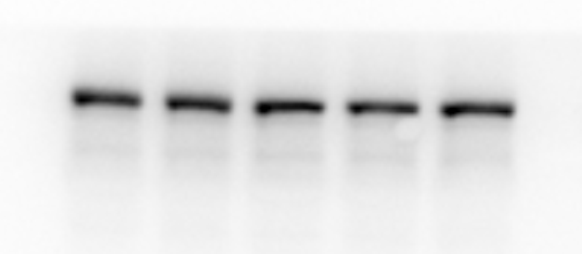


p-STING


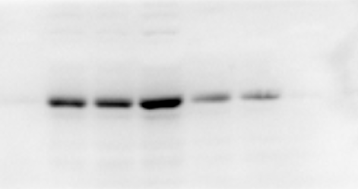


STING


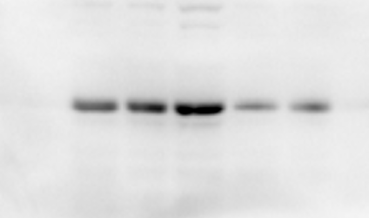


GAPDH


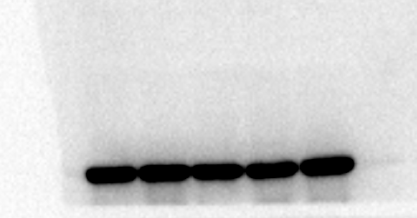


Figure 6a

p-TBK1


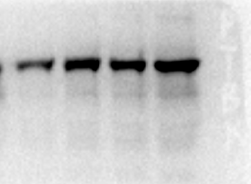


TBK1


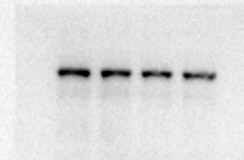







p-STING


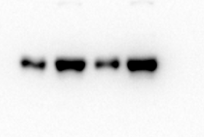







Figure 6c

p-TBK1


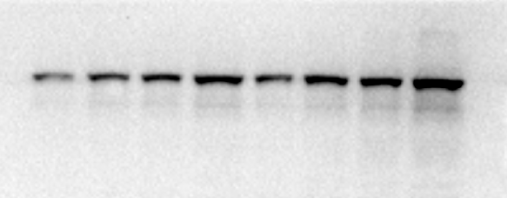


TBK1


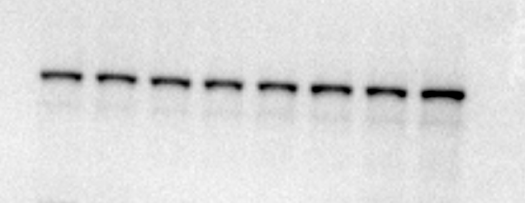







p-STING


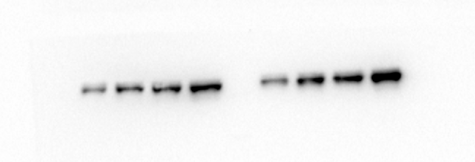









Figure 6e

p-TBK1


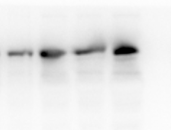


TBK1


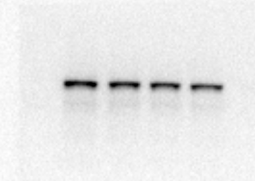


p-TBK1


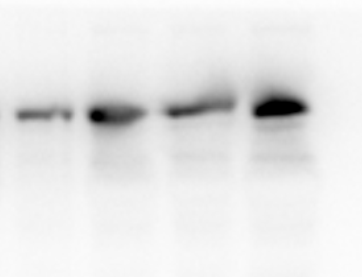











Figure 6i

p-TBK1


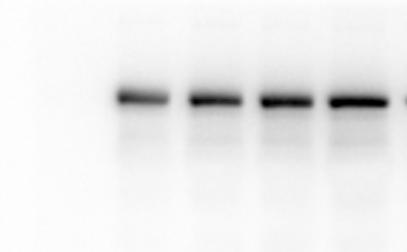


TBK1


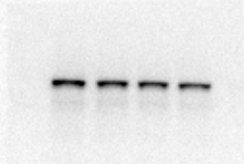


IRF3


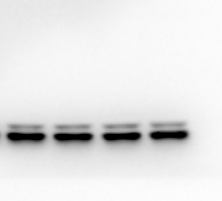







p-STING


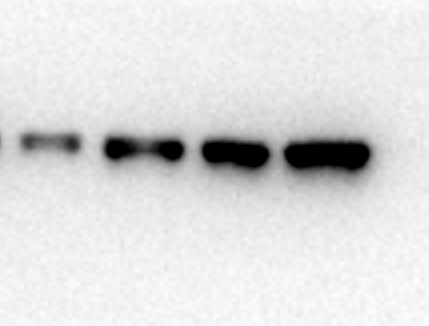





Figure 7c

p-AKT-T308


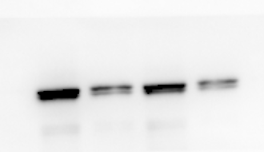









Figure 7e

p-AKT-T308


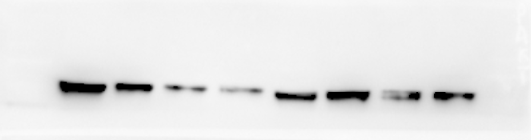


p-STING


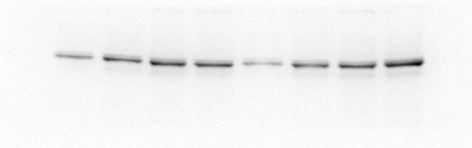







STING


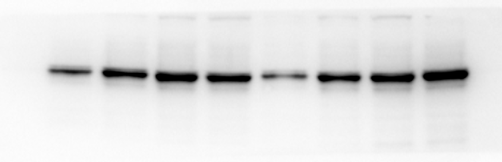




Figure 7g

p-STING


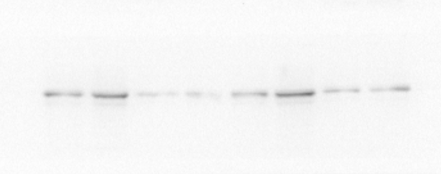


p-AKT-T308


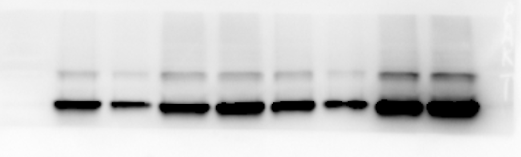







AKT


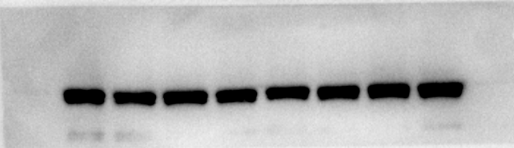




STING


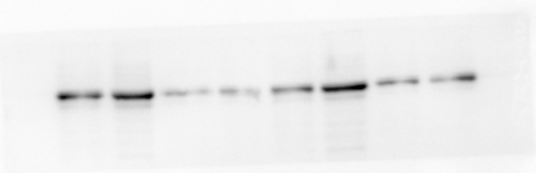




Figure S1b

GAPDH


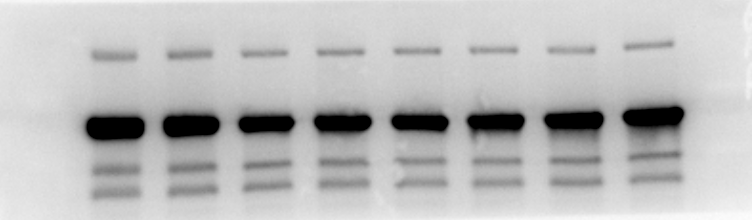


SGLT2

Figure S1c

GAPDH

TRIM21

Figure S3e

GAPDH

STING

Figure S3j

STING

SGLT2

GAPDH

Figure S4e

STING

GAPDH
